# Effect of serum concentrations of IL-6 and TNF-α on brain structure in anorexia nervosa: a combined cross-sectional and longitudinal study

**DOI:** 10.1038/s41386-024-01836-z

**Published:** 2024-03-09

**Authors:** Fabio Bernardoni, Friederike Tam, David M. Poitz, Inger Hellerhoff, Dominic Arold, Daniel Geisler, Frances Lemme, Johanna Keeler, Kerstin Weidner, Carmine Pariante, Veit Roessner, Joseph A. King, Stefan Ehrlich

**Affiliations:** 1 Translational Developmental Neuroscience Section, Division of Psychological and Social Medicine and Developmental Neuroscience, Faculty of Medicine, Technische Universität Dresden, Dresden, Germany; 2 Eating Disorder Research and Treatment Center, Department of Child and Adolescent Psychiatry, Faculty of Medicine, Technische Universität Dresden, Dresden, Germany; 3 University Hospital Carl Gustav Carus, Institute of Clinical Chemistry and Laboratory Medicine, Technische Universität Dresden, Dresden, Germany; 4 Department of Psychological Medicine, Institute of Psychiatry, Psychology & Neuroscience, King’s College London, London, UK; 5 Department of Psychotherapy and Psychosomatic Medicine, Faculty of Medicine, Technische Universität Dresden, Dresden, Germany; 6 Department of Child and Adolescent Psychiatry, Faculty of Medicine, Technische Universität Dresden, Dresden, Germany

**Keywords:** Brain, Neuroimmunology

## Abstract

Previous studies of brain structure in anorexia nervosa (AN) have reported reduced gray matter in underweight patients, which largely normalizes upon weight gain. One underlying biological mechanism may be glial cell alterations related to low-grade inflammation. Here, we investigated relationships between brain structure as measured by magnetic resonance imaging and serum concentrations of two pro-inflammatory cytokines (interleukin-6 and tumor necrosis factor alpha) cross-sectionally in 82 underweight adolescent and young adult female patients (mean age 16.8 years; 59 of whom were observed longitudinally after short-term weight restoration; mean duration 2.8 months), 20 individuals long-term weight-recovered from AN (mean age 22.7 years) and 105 healthy control (HC) participants (mean age 17.2 years). We measured cortical thickness, subcortical volumes and local gyrification index, a measure of cortical folding. In contrast to most previous studies of cytokine concentrations in AN, we found no cross-sectional group differences (interleukin-6: *p* = 0.193, tumor necrosis factor alpha: *p* = 0.057) or longitudinal changes following weight restoration (interleukin-6: *p* = 0.201, tumor necrosis factor alpha: *p* = 0.772). As expected, widespread gray matter reductions (cortical thickness, subcortical volumes, cortical folding) were observed in underweight patients with AN compared to HC. However, we found no evidence of associations between cytokine concentrations and structural brain measures in any participant group. Furthermore, longitudinal changes in cytokine concentrations were unrelated to changes in gray matter. In conclusion, we did not identify any association between (sub-)inflammatory processes and structural brain changes in AN. Future studies are needed to elucidate which other factors besides nutritional status may contribute to brain morphological alterations.

## Introduction

The factors contributing to the development and maintenance of anorexia nervosa (AN), a life-threatening eating disorder with a typical onset in adolescence, are not fully understood. For instance, accumulating evidence points toward a low-grade inflammation with increased pro-inflammatory cytokines in patients with AN [1]. This might be an underlying mechanism of glial cell alterations in AN, which may in turn contribute to the well-documented substantial reduction of brain mass including cortical thickness (CT) in the acute state of the disorder. However, to date, no study has systematically investigated the connection between cytokines and CT reduction in AN.

Underlying mechanisms of low-grade inflammation found in AN are still largely unclear, but a few hypotheses have been suggested. Elevated cortisol concentrations in the underweight state, excessive physical activity and the altered gut microbiome [2–4] may modulate intestinal permeability and allow bacteria/bacterial parts to traverse into peripheral circulation inducing low-grade inflammation [5–7]. In line with this, two large meta-analyses showed elevated blood concentrations of pro-inflammatory cytokines in acute AN, with the largest number of samples for interleukin-6 (IL-6) and tumor necrosis factor alpha (TNF-α) [1, 8]. Both IL-6 and TNF-α may facilitate a prolonged response of the pro-inflammatory nuclear factor kappa beta, and TNF-α may increase vascular permeability and thus contribute to the “leaky gut” in AN [9]. Furthermore, animal models have provided evidence for anorexigenic effects of IL-6 and TNF-α [8]. IL-6 interacts with the appetite-regulating hormone leptin and regulates multiple aspects of metabolism [10]. Additionally, preliminary evidence from human studies proposes a link between exercise-induced IL-6 release and the transient suppression of appetite and food intake [11, 12]. Also, TNF-α promotes the production of anorexigenic peptides [8] and may act as a modulator of energy storage in adipocytes [13]. Data on longitudinal trajectories of cytokine concentrations during weight restoration are scarce [8, 14].

In the underweight state of AN, patients show widespread and substantial structural gray matter (GM) changes, such as a reduction of CT and cortical folding [15, 16]. After long-term weight recovery, previous studies have shown these GM changes to mostly normalize [17]. Our own findings point toward a rapid normalization of CT and cortical folding during short-term weight restoration therapy of ~3 months [15, 18, 19]. However, cortical folding has a different genetic origin [20] and pattern of alterations compared to CT [15]. Since cortical surface area alterations in AN seem smaller compared to CT (~1.6% vs. 6.4%) and the interregional pattern of cortical volume alterations largely overlaps that observed for CT [18], GM volume changes seem to be driven by CT changes. The underlying mechanisms of structural GM changes remain elusive, with hypothesized mechanisms including fluid shifts due to changes in oncotic pressure, the loss, damage or remodeling of glia and/or neurons and hormonal changes [17]. Furthermore, inflammatory processes may contribute to changes in brain structure. While evidence for this is still scarce in AN, previous studies in other psychiatric disorders support such a link [21–29]. For instance, findings in major depressive disorder, which is one of the most common comorbidities in AN, give rise to the hypothesis that alterations in brain structure may be due to the neurotoxic effects of increased inflammation [21]. In AN, using a virtual histology analysis, we found that brain regions with a high expression of genes specific to oligodendrocytes are most affected by the reductions [18]. Pro-inflammatory factors such as circulating cytokines could lead to an activation of astrocytes, of which a subgroup may promote toxicity for neurons and oligodendrocytes [30]. Consistent with this, blood concentrations of the neuronal damage marker neurofilament light (NF-L) and of glial fibrillary acidic protein (GFAP), which has been associated with astroglial injury, are elevated in AN [31, 32], and NF-L has been linked to cortical thinning in AN [33]. The loss or dysfunction of oligodendrocytes might result in demyelination and eventually neuronal death.

Therefore, the aim of the present study was to investigate the potential relationship between inflammatory processes and dynamic structural GM changes in AN. To this end, we assessed serum concentrations of TNF-α and IL-6 as well as CT, local gyrification index (lGI), a measure of cortical folding, and volumes of subcortical gray matter nuclei in (1) young patients with AN with a relatively short duration of illness, both when underweight and after short-term weight restoration (lasting ~3 months), (2) individuals long-term recovered from AN (for at least 6 months) and (3) healthy control (HC) participants. Based on previous research [1, 8], we expected IL-6 and TNF-α to be elevated in the acute state of AN as a sign of low-grade inflammation, and that altered cytokine concentrations would be linked to reduced CT, IGI and volumes of subcortical nuclei in underweight patients with AN. Furthermore, we hypothesized a decrease in IL-6 and TNF-α over the course of short-term weight restoration to predict normalization of structural GM measures. Long-term weight-recovered individuals were included to differentiate alterations related to undernutrition (state markers) from alterations that may confer vulnerability toward AN (trait markers) or long-term effects of the disorder. Furthermore, we explored associations of cytokine concentrations with markers of disorder severity, i.e., psychopathology, body mass index standard deviation scores (BMI-SDS) and plasma leptin concentrations. Plasma leptin concentrations served as a marker of undernutrition as they reflect energy stores and positively adipose tissue mass [34].

## Methods and materials

### Participants

A total of 217 female participants took part in the study: (85 underweight patients with AN (acAN, 12–28 years old), 24 individuals long-term weight-recovered from AN (recAN, 17–29 years old) and 108 healthy control participants (HC, 12–28 years old). All acAN participants were admitted to intensive treatment of specialized eating disorder programs at the child and adolescent psychiatry and psychosomatic medicine department of a university hospital. All acAN underwent the first assessments within 96 h of beginning nutritional rehabilitation (acAN-TP1). Sixty-three acAN were re-examined after partial weight restoration (at least 12% increase in body mass index (BMI) as inclusion criterion, range: 14.1–45.8%; acAN-TP2). After applying quality control procedures (see below), 266 scans were used in the analysis (82 acAN-TP1, 59 acAN-TP2, 20 recAN, 105 HC).

Diagnoses of eating disorders and other information regarding potential confounding variables (e.g., medication, comorbidities, smoking) were obtained using the expert form of the Structured Interview for Anorexia and Bulimia Nervosa (SIAB-EX) [35], supplemented with our own semi-structured interview. Interviews were adapted to DSM-5 criteria and carried out by clinically experienced research assistants under the supervision of a child and adolescent psychiatrist. A diagnosis of AN required a BMI below the 10th age percentile (if <15.5 years old) or below 17.5 kg/m² (if >15.5 years old). RecAN participants had to have previously met diagnostic criteria for AN, maintained a BMI > 18.5 kg/m² (or the 10th age percentile if <18 years), had regular menstruation and not engaged in significant restrictive eating behavior (or binging/purging) for 6 months prior to study participation. HC participants had to have a normal BMI (18.5–29 kg/m^2^ or >10th and <95th age percentile if <18 years), regular menstruation and have no history of psychiatric illness and were recruited through advertisement among middle/high school and university students. For exclusion criteria and comorbid diagnoses, see Supplementary 1.1. All protocols received ethical approval by the local Institutional Review Board, and all participants (or legal guardians) gave written informed consent.

### Clinical measures

In addition to the evaluation with the SIAB-EX [35], we assessed eating disorder-related psychopathology with the Eating Disorder Inventory-2 (EDI-2) [36], depressive symptoms with the Beck Depression inventory-II (BDI-II) [37] and anxiety symptoms using the anxiety scale of the revised Symptom Checklist 90 (SCL-90-R) [38]. IQ was estimated with the Wechsler Adult Intelligence Scale (if age > 16 years) [39] or the Wechsler Intelligence Scale for Children (if age ≤ 16 years) [40]. BMI-SDS [41] were computed to provide an age-corrected index.

### Blood sample preparation and analysis of cytokines and leptin

Venous blood samples were collected between 7 and 9 a.m. after an overnight fast. To yield blood serum for IL-6 and TNF-α assessment, samples were left to clot for 30 min at 6–8 °C and then centrifuged (2500 × *g*, 15 min, 5 °C). To yield plasma for leptin assessment, aprotinin was added during blood sampling to prevent protein degradation by serine proteases and samples were immediately centrifuged (2500 × *g*, 15 min, 5 °C). All samples were aliquoted immediately after centrifugation and stored at −80 °C. Serum IL-6 and TNF-α concentrations were measured in duplicate with commercially available high-sensitivity enzyme immunoassays (IL-6: IBL International GmbH, Hamburg, Germany; TNF-α: R&D Systems Inc., Minneapolis, MN, USA). Participants were excluded from analyses if the coefficient of variation between duplicate measurements exceeded 20% for either IL-6 or TNF-α (2 recAN, 2 HC). Only two IL-6 measurements (none for TNF-α) were below the limit of detection of 0.03 pg/ml and were imputed as $\frac{{{{{{{\rm{limit}}}}}}\; {{{{{\rm{of}}}}}}\; {{{{{\rm{detection}}}}}}}}{\sqrt{2}}$. Plasma leptin concentrations were measured using a commercially available enzyme-linked immunosorbent assay (ELISA, BioVendor Research and Diagnostic Products, Brno, Czech Republic). For leptin, 20 acAN-TP1 measurements were below the limit of detection and were imputed using a quantile regression multiple imputation approach for left-censored missing data [42] (Supplementary 1.2).

### Structural MRI acquisition and image data processing

All participants underwent MRI between 8 and 9 a.m. following an overnight fast. High-resolution three-dimensional T1-weighted structural scans were acquired on Siemens Magnetom Trio 3T Scanner with a magnetization-prepared rapid gradient-echo (MP-RAGE) sequence using the same parameters as in our previous studies [19, 43] (Supplementary 1.3). Reconstruction of the cerebral cortex was accomplished automatically with FreeSurfer 7.1 [44, 45] (Supplementary 1.4), followed by standardized quality control by trained raters (Supplementary 1.5). Scans with artefacts that exert a significant influence on parcellation (mainly dura inclusions in the pial surface) were excluded from the analysis (3 acAN-TP1, 4 acAN-TP2, 2 recAN and 1 HC). All images underwent further longitudinal preprocessing with the FreeSurfer longitudinal stream [46]. At each vertex on the resulting tessellated pial surface, CT [47] and lGI [48] were measured and smoothed using a Gaussian kernel with a full-width-at-half-maximum of 10 mm, and volumes of 8 subcortical gray-matter nuclei were measured (Supplementary 1.6).

### Statistical analyses

Due to deviations from normality, IL-6, TNF-α and leptin were log_e_-transformed before parametric tests were applied. Spearman correlation coefficients were calculated to investigate possible associations between cytokine concentrations and clinical variables including BMI-SDS, leptin concentrations and psychopathology. Statistical significance was defined as *p* < 0.05.

Statistical modeling of CT and lGI was based on a linear mixed effect model implemented using Freesurfer mixed effect tools [49]. We aimed to test whether changes in cytokine concentrations explain variance in GM changes not already accounted for by changes in BMI-SDS [15, 19]. Given the distinct developmental trajectories of the three investigated measures [50], age was included as a linear covariate. In sum, we modeled CT and lGI as:

$${{CT}},{{lGI}}=A+{\Delta }_{{{{{{{\rm{recAN}}}}}}}}+{\Delta }_{{{{{{{\rm{acAN}}}}}}}}+{B}_{{{{{{{\rm{acAN}}}}}}}}\left({b}_{t}-{b}_{{{{{{{\rm{TP}}}}}}}2}\right)+{D}_{{{{{{{\rm{acAN}}}}}}}}\left({d}_{t}-{d}_{{{{{{{\rm{TP}}}}}}}2}\right)+C\,{{{{{{\rm{age}}}}}}}$$

where ${b}_{t}$ and ${d}_{t}$ represent BMI-SDS and the log_e_-transformed serum concentrations of the cytokines IL-6 or TNF-α, respectively, at timepoint *t* (TP1 or TP2). For subcortical volumes, we included total intracranial volume as an additional covariate.

This approach allowed us to examine cross-sectional group comparisons (acAN-TP1 vs. HC, recAN vs. HC), and longitudinal changes associated with BMI-SDS and cytokine concentrations [19]. Compared to the use of repeated measures to examine longitudinal changes, this approach allowed us to include also participants with single point data, which lead to a better estimation of the variance. To assess the role of AN subtype and of comorbidities, we repeated the above analyses by excluding participants with AN of binge/purge subtype and with psychiatric and somatic comorbidities (including very mild/local infections), respectively. Furthermore, to control for possible effects of smoking on IL-6 and TNF-α [51], and non-linear age effects, we included the variables “current number of cigarettes per day” and “ever-smoking status”, and age^2^ as covariates in supplementary analyses [52]. To investigate relationships between cytokine levels and brain structure in each group, we used general linear models with cytokine levels and age as covariates. We corrected for multiple comparisons using a false discovery rate *q* < 0.05 for each hemisphere unless otherwise noted, and across cytokine type using the Bonferroni criterium. Further analysis details are provided in Supplementary 1.7, 1.8. Statistical analyses were performed using Matlab, R [53] and IBM SPSS Statistics for Windows, version 27.0 (IBM Corp., Armonk, NY).

## Results

### Demographic and clinical characteristics

The demographic and clinical data are presented in Table 1. As expected, acAN-TP1 patients had lower BMI-SDS and leptin concentrations and higher symptom levels (EDI-2, BDI-II, SCL-90-R anxiety scale) than HC, which improved after short-term weight restoration (except for anxiety). Most notably, BMI increased by mean(SD) = 27.0(8.4)% during weight-restoration treatment (duration mean(SD) = 2.77(0.84) months, range 35–155 days), but was still under the age-appropriate average in acAN-TP2. RecAN were slightly older and still had some residual psychological symptoms. Furthermore, recAN consumed a higher number of cigarettes per day than acAN.

**Table 1:** Demographic and clinical data.

	Observation time point		Longitudinal change	Control samples		Cross-sectional analysis			
	acAN-TP1 (*N* = 82)	acAN-TP2 (*N* = 59)	acAN-TP2–acAN-TP1 (*N* = 59)	recAN (*N* = 20)	HC (*N* = 105)	*F*/*H*	df	*p*	post hoc tests
Age	16.8(3.2)	16.7(2.2)	0.23(0.07)***	22.7(3.4)	17.2(3.5)	25.47	204	<0.001	recAN > acAN-TP1*** recAN > HC***
BMI (kg/m^2^)	15.0(1.2)	19.2(1.0)	4.0(1.0)***	21.6(1.5)	20.6(2.0)	279.71	204	<0.001	acAN-TP1 < recAN,HC***
BMI-SDS	−3.1(1.2)	−0.7(0.6)	2.3(0.7)***	−0.23(0.54)	−0.13(0.66)	257.57	204	<0.001	acAN-TP1 < recAN,HC***
Minimal lifetime BMI (kg/m²)	14.6(1.3)	n/a	n/a	14.6(1.6)	19.5(1.8)	233.65	192	<0.001	HC > acAN-TP1,recAN***
IQ	111(12)	n/a	n/a	111.9(10.5)	111.68(9.07)	0.03	197	0.975	All not significant
BDI-II total score	24.1(10.8)	15.1(11.2)	7.8(10.5)***	8.1(8.0)	4.8(5.6)	129.19	202	<0.001	acAN-TP1 > recAN,HC***
EDI-2 total score	213.0(45.0)	195.1(52.7)	12.4(40.9)*	154.7(34.9)	138.1(27.2)	98.55	198	<0.001	acAN-TP1 > recAN,HC***
SCL-90-R anxiety scale	1.0 (0.8)	0.7 (0.7)	−0.1 (0.4)	0.4 (0.4)	0.3 (0.5)	37.03	202	<0.001	acAN-TP1 > recAN,HC***
Plasma leptin (μg/l)	1.46(1.75)	12.05(6.37)	10.56(5.80)***	9.37(5.01)	12.12(8.95)	136.54	202	<0.001	acAN-TP1 < recAN, HC***
Cigarettes per day	0.1(0.9)	0.2(1.3)	0.03(0.18)	2.1(4.6)	0.3(1.6)	9.92	2	0.007	acAN-TP1 < recAN**

Mean (standard deviation) are presented for each group in a separate column. Group differences between acAN-TP1, recAN and HC were tested with a one-way ANOVA followed by Tukey’s post hoc test (*p < 0.05, **p < 0.01, ***p < 0.001), and longitudinal differences between acAN-TP1 and acAN-TP2 were tested with dependent samples *t*-tests. Due to deviations from normality, leptin concentrations and SCL-90R anxiety scores were log_e_-transformed before parametric tests were applied. Due to the right-skewed distribution of the variable cigarettes per day, non-parametric tests were used (Kruskal–Wallis test for cross-sectional analysis followed by post hoc Dunn-tests, Wilcoxon signed-rank test for longitudinal analysis). Regarding ever-smoking status, 10/82 acAN-TP1, 7/59 acAN-TP2, 11/20 recAN and 24/105 HC reported currently smoking cigarettes or to having smoked cigarettes in the past. The mean age of onset of AN in the acAN sample was 14.6(2.8) years and the mean duration of illness was 14(20) months. recAN have been weight-recovered for an average of 60(43) months. 71 acAN were of the restrictive subtype (87%) and 11 (13%) were of the binge/purge subtype. 12 (60%) recAN were of the restrictive subtype during illness and 8 (40%) were of the binge/purge subtype. In the acAN group, 80/82 identified as White and 2/82 identified as Asian. In the recAN group, 20/20 identified as White. In the HC group, 103/105 identified as White, 1/105 identified as African and 1/105 identified as Asian.

*acAN-TP1* acutely underweight participants with AN at timepoint 1 (admission), *acAN-TP2* participants with AN at timepoint 2 (after short-term weight restoration), *HC* healthy control participants, *recAN* individuals long-term weight-recovered from AN, *BDI-II* Beck Depression Inventory-II, *BMI-SDS* body mass index-standard deviation score, *EDI-2* Eating Disorder Inventory-2, *SCL-90R* Symptom Checklist-90-Revised.

### Cytokines

**Fig. 1:**
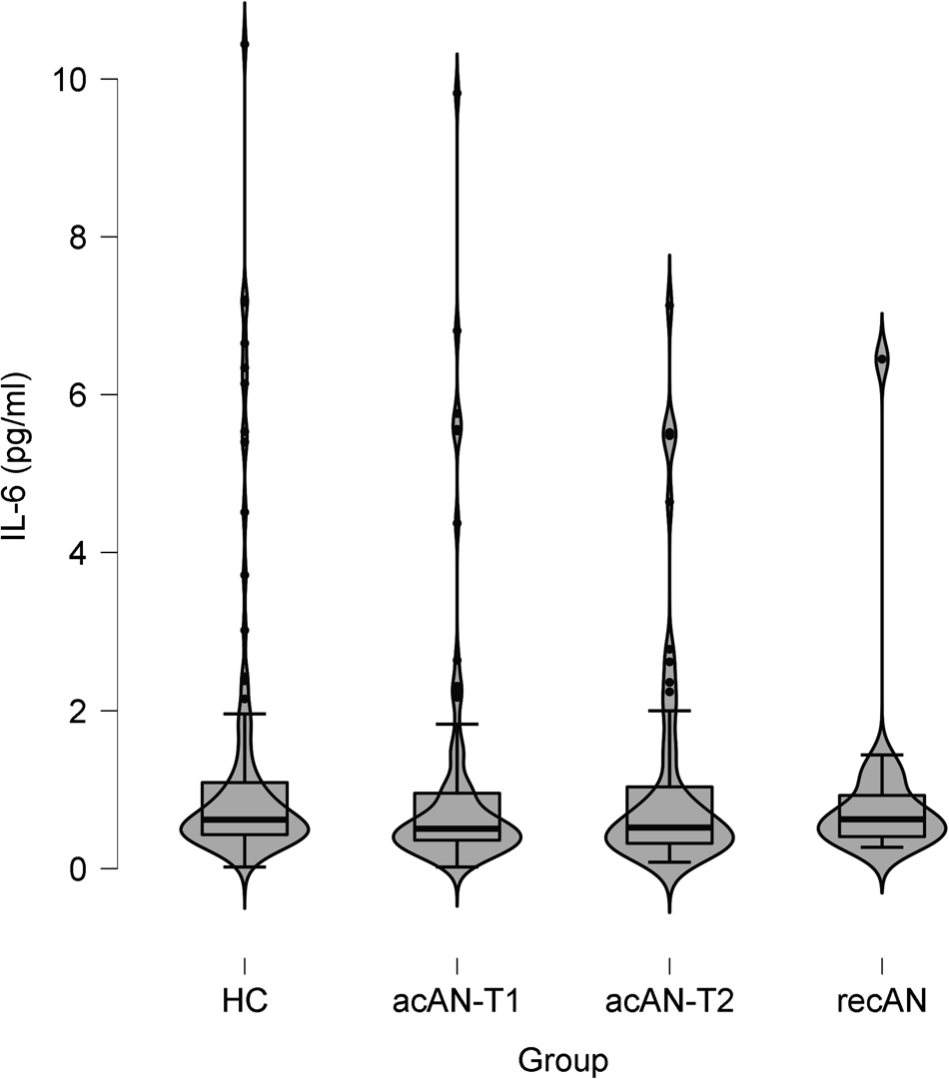
Serum IL-6 concentrations. Violin plots showing the median, upper and lower quartile, outliers (depicted as circles, values deviating more than 1.5 times the interquartile range from the upper or lower quartile) and the kernel probability density of the data. Serum IL-6 concentrations (pg/ml): HC: Median = 0.62, interquartile range = 0.70, range = <0.03–10.44; acAN-T1: Median = 0.51, interquartile range = 0.61, range = <0.03 to 9.82; acAN-T2: Median = 0.52, interquartile range = 0.75, range = 0.08–7.13; recAN: Median = 0.63, interquartile range = 0.63, range = 0.27–6.45. The figure was created with JASP [65]. acAN-TP1 acutely underweight participants with AN at timepoint 1 (admission), acAN-TP2 participants with AN at timepoint 2 (after short-term weight restoration), HC healthy control participants, recAN individuals long-term weight-recovered from AN.

**Fig. 2:**
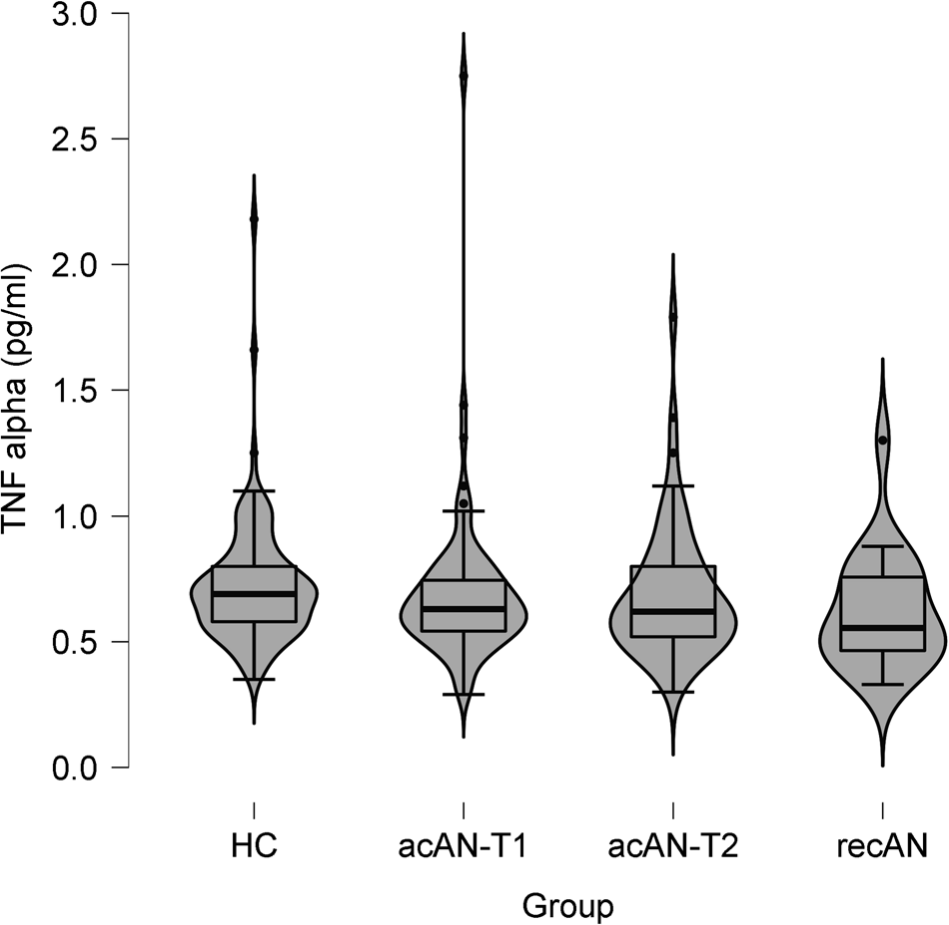
Serum TNF-α concentrations. Violin plots showing the median, upper and lower quartile, outliers (depicted as circles, values deviating more than 1.5 times the interquartile range from the upper or lower quartile) and the kernel probability density of the data. Serum TNF-α concentrations (pg/ml): HC: Median = 0.69, interquartile range = 0.23, range = 0.35–2.18; acAN-T1: Median = 0.63, interquartile range = 0.22, range = 0.29–2.75; acAN-T2: Median = 0.62, interquartile range = 0.28, range = 0.30–1.79; recAN: Median = 0.56, interquartile range = 0.32, range = 0.33–1.30. The figure was created with JASP [65]. acAN-T1 acutely underweight participants with AN at timepoint 1 (admission), acAN-T2 participants with AN at timepoint 2 (after short-term weight restoration), HC healthy control participants, recAN individuals long-term weight-recovered from AN.

One-way ANOVAs revealed no significant differences between the acAN-TP1, recAN and HC groups in IL-6, *F*(2,204) = 1.66, *p* = 0.193 (Fig. 1), or TNF-α concentrations, *F*(2,204) = 2.91, *p* = 0.057 (Fig. 2). These results did not change when covarying for age (Supplementary 2.1) or smoking (Supplementary 2.2), or using non-parametric tests (Supplementary 2.3). Bayesian analysis provided anecdotal evidence in support for the null hypothesis (Supplementary 2.4). In acAN, paired samples *t*-tests showed no change of serum concentrations over the course of short-term weight restoration for IL-6, *t*(58) = −1.30, *p* = 0.201 (Fig. 1), or TNF-α, *t*(58) = −0.29, *p* = 0.772 (Fig. 2), and Bayesian analysis provided moderate evidence in support for the null hypothesis (Supplementary 2.4). Both the cross-sectional and the longitudinal analyses yielded the same results when excluding participants with psychiatric and somatic comorbidities (Supplementary 2.5) or of the AN binge/purge subtype (Supplementary 2.6). In the correlation analysis, no link between cytokine concentrations and BMI-SDS, leptin or measures of psychopathology (EDI-2, BDI-II total scores, SCL-90-R anxiety scale) emerged after correcting for multiple comparisons (Supplementary Table S1).

### Structural MRI measures

**Fig. 3:**
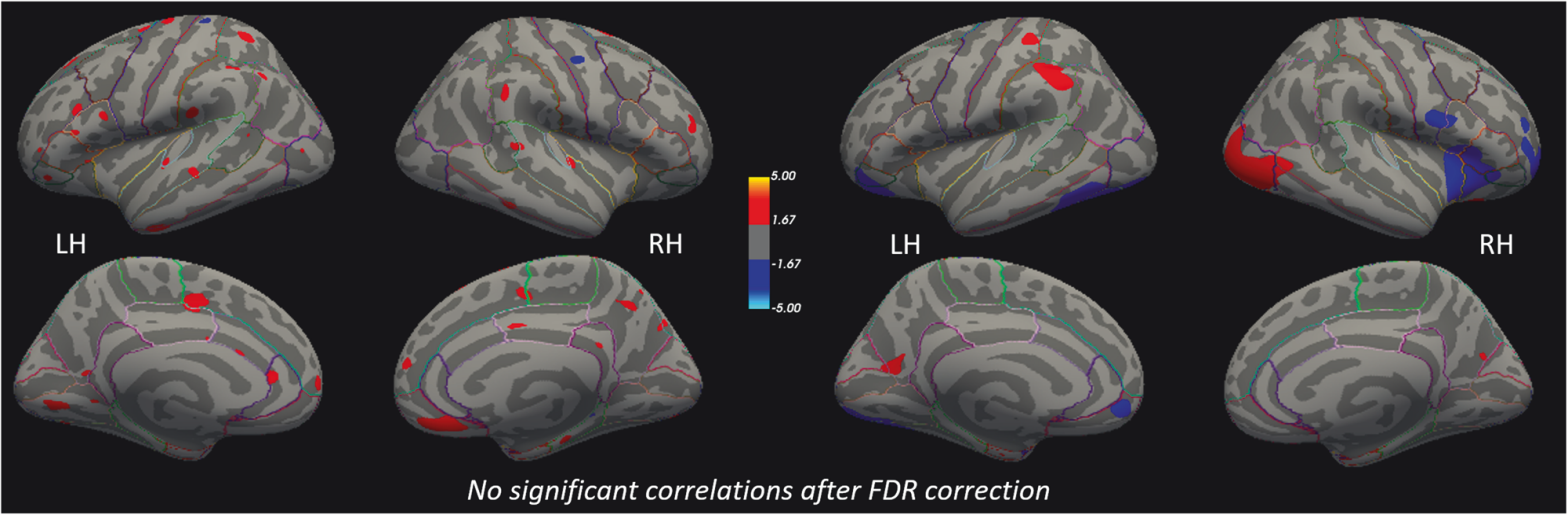
No significant associations between longitudinal changes in cortical structure (acAN-TP2-acAN-TP1) and changes in serum concentrations of the IL-6 cytokine. Left: Associations with CT changes. Right: Associations with lGI changes. Uncorrected statistical maps (*p* < 0.05) plotted on the inflated surface of the standard average subject and displaying regions in which differences in CT or lGI between acAN-TP2 and acAN-TP1 were associated with changes in IL-6. The color scale shows *p* values expressed as −log_10_(*p*). Warm colors indicate a positive correlation between changes in IL-6 concentrations and CT or lGI. After FDR-correcting for multiple comparisons (*q* < 0.05) across each hemisphere and Bonferroni correcting across cytokine type, these associations were not significant. LH left hemisphere, RH right hemisphere. Colored outlines correspond to anatomical labels of the Desikan-Killiany atlas [44].

**Fig. 4:**
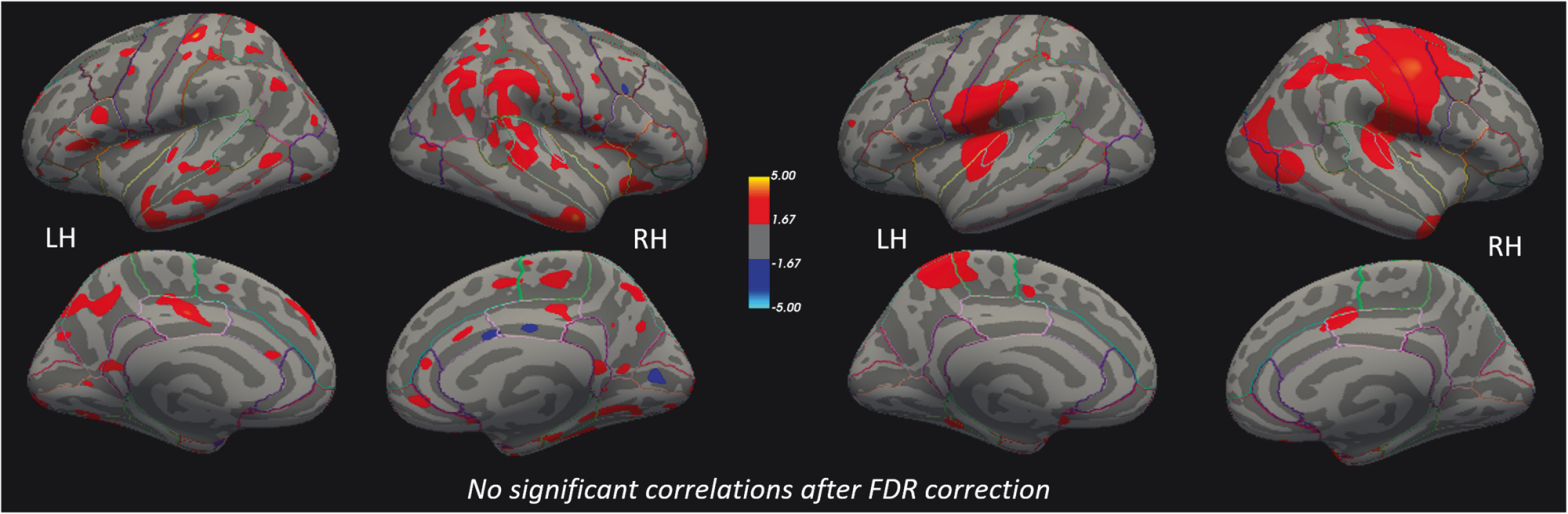
No significant associations between longitudinal changes in cortical structure (acAN-TP2-acAN-TP1) and changes in serum concentrations of the TNF-α cytokine. Left: Associations with CT changes. Right: Associations with lGI changes. Uncorrected statistical maps (*p* < 0.05) plotted on the inflated surface of the standard average subject and displaying regions in which differences in CT or lGI between acAN-TP2 and acAN-TP1 were associated with changes in TNF-α. The color scale shows *p* values expressed as −log_10_(*p*). Warm colors indicate a positive correlation between changes in TNF-α and CT or lGI. After FDR-correcting for multiple comparisons (*q* < 0.05) across each hemisphere and Bonferroni correcting across cytokine type, these associations were not significant. LH left hemisphere, RH right hemisphere. Colored outlines correspond to anatomical labels of the Desikan-Killiany atlas [44].

Replicating our previous findings [15, 19], we observed a widespread reduction of CT and lGI in acAN at baseline (acAN-TP1) relative to HC, and longitudinal increases related to partial weight restoration (Figs. S1 and S2). No significant differences between recAN and HC were revealed, neither in CT nor in lGI. However, lGI was still reduced in some areas of the cortex in acAN-TP2 (Fig. S3). Contrary to our motivating hypothesis, neither CT, nor lGI were associated with IL-6 or TNF-α concentrations in any of the groups considered (acAN-TP1, acAN-TP2, recAN, HC, Figs. S4–S11). Furthermore, longitudinal changes in IL-6 (Fig. 3) or TNF-α (Fig. 4) were not associated with longitudinal changes in either CT, lGI, or subcortical volumes (Table S3). This was also the case when (1) excluding participants with psychiatric and somatic comorbidities (Figs. S12 and S13) or of the binge-purge subtype from the AN groups (Figs. S14 and S15). No effects of age^2^ (Fig. S16) and “ever-smoking status” (Fig. S17) were evident, but the “current number of cigarettes per day” was associated with CT (but not lGI) alterations in the motor and pre-motor cortex (Fig. S18). However, even when accounting for these additional covariates, longitudinal changes in IL-6 (Fig. S19) or TNF-α (Fig. S20) were still not associated with longitudinal changes in either CT or lGI.

## Discussion

In the present study, we measured serum concentrations of the pro-inflammatory cytokines IL-6 and TNF-α in conjunction with sMRI scans cross-sectionally in a large sample of underweight adolescents and young adults with AN, individuals long-term weight-recovered from AN, and HC. Most patients with AN were re-examined after short-term weight restoration. To the best of our knowledge, our study was the first to investigate the potential relationship between peripheral inflammatory markers and structural brain changes in AN. Contrary to our hypothesis of a low-grade systemic inflammation in the acute state of the disorder, there were no significant group differences in cytokine concentrations between acAN-TP1, recAN, and HC in our adolescent and young adult sample. Also, the longitudinal analysis indicated no change in cytokine concentrations over the course of short-term weight restoration (mean duration 2.8 months). We replicated previous findings of widespread cortical thinning and reduced cortical folding (assessed by IGI) and reduced volumes of subcortical GM nuclei in acAN-TP1 compared to HC, which were largely reversed already after short-term weight restoration. However, no measure of GM structure was linked to cytokine concentrations in any of the groups. Furthermore, there were no associations between longitudinal changes in CT, lGI, or subcortical volumes and changes in cytokine concentrations. These findings were confirmed in supplementary analyses controlling for non-linear effects of age and smoking as well as excluding participants with the AN binge/purge subtype and with psychiatric and somatic comorbidities.

Our null finding in the cross-sectional analysis of cytokines, i.e., no differences in concentrations between the groups, is partially consistent with the findings of prior studies. While two large meta-analyses indicated that patients with AN had elevated blood concentrations of IL-6 and TNF-α [1, 8], more recent studies presented heterogenous results [54–57]. For instance, a study on a large adult sample reported normal IL-6 concentrations in patients with AN compared to HC [54]. Keeler et al. reported normal TNF-α and reduced IL-6 concentrations in AN patients, while carefully controlling for potentially confounding variables, including, as in the current study age, smoking status and use of psychopharmacological medications [55]. Of note, one meta-analysis [1] highlighted the moderate to high levels of heterogeneity among previous studies, suggesting that variation in clinical characteristics such as age at onset, illness duration, or stage of treatment may be contributing factors. Additionally, they identified age as a factor influencing IL-6 concentrations [1]. This may be especially important because the participants in our study were younger than those included in the meta-analysis (mean age: our study sample: 16.8 years, Solmi et al. [8]: 21.7 years, Dalton et al. [1]: 21.4 years). Also, it has been speculated that in the early stages of AN, higher endogenous opioid release, which may positively reinforce fasting, could lead to a lower inflammatory response or even an immunosuppressive state [58]. This would be consistent with research suggesting that intermittent fasting leads to anti-inflammatory effects as well as to a reduction of anxiety and depressive symptoms [59–61]. Our data suggests that young patients with a relatively short duration of illness (mean: 14 months) may not show significant signs of systemic inflammation as assessed by serum TNF-α and IL-6. This is partly in line with a study in adolescents with AN, which found elevated TNF-α but reduced IL-6 concentrations [56]. However, a study in adolescents (mean age 14.9 years) with short illness duration (mean: 14 months) reported elevated TNF-α plasma concentrations [62]. In another young transdiagnostic sample including male and female patients with AN and other eating disorders (mean age 17.7 years for female participants), patients showed normal serum TNF-α levels and lower concentrations of IL-6 and the pro-inflammatory cytokine IL-1 beta [58].

Furthermore, recAN showed no group differences in cytokines when compared to HC, which is consistent with the findings from the largest study to date that included recovered patients [54], while another study reported reduced TNF-α in recovered individuals in comparison to HC but no group difference for IL-6 [55]. Our finding of no longitudinal change in cytokine concentrations over the course of short-term weight restoration is partly in line with previous findings: The meta-analysis from Solmi et al. [8] as well as a more recent study [14] found no significant difference in IL-6 concentrations before and after weight gain, while for TNF-α no difference [8] or a decrease from baseline to reassessment [14] were reported.

Additionally, our exploratory correlational analyses did not show any correlation between cytokine concentrations and indicators of undernutrition (BMI-SDS, leptin concentrations) or psychopathology for any of the groups. The heterogeneity of the published results emphasizes the complexity of possible inflammatory processes in AN and the need for further large-scale studies to identify potential patient subgroups prone to systemic inflammation [54].

The absence of any association between measures of gray matter (GM) and serum concentrations of IL-6 or TNF-α in any of the groups may be attributed to the lack of peripheral inflammation in the sample. None of the study participants exhibited cytokine concentrations indicative of systemic inflammation, as all values were below established clinical thresholds. Therefore, it seems unlikely that the marked and widespread CT reductions observed in underweight adolescent and young females with AN are due to (sub)-inflammatory processes. Recently, higher serum concentrations of the neuronal damage marker NF-L were found to be associated with lower CT in several brain regions in a study sample of acAN-TP1 similar to ours, which implies axonal damage as a potential underlying mechanism of cortical thinning [33]. However, an exploratory analysis in part of this sample did not find a correlation between serum concentrations of NF-L and IL-6 or TNF-α (Supplementary Table S2), again rendering it unlikely that (sub)-inflammatory processes that can be captured with IL-6 or TNF-α explain GM decreases in acute AN. While various other mechanisms of CT reduction have been hypothesized (e.g., the loss, damage or remodeling of glia and/or neurons, see Introduction), a recent study using a virtual histology approach highlighted that the most vulnerable regions are those with a higher density of energetically demanding cells in adolescents and young female patients [18]. This further supports longitudinal findings showing a strong association between GM changes in AN and nutritional status. However, these findings were mostly obtained in adolescents and young female participants, and the recovery was slower in older participants [18]. Since also the heterogeneity of findings regarding cytokine concentrations in different eating disorder patient populations seems to be related to age effects [1], future studies should investigate whether sub-inflammatory processes might play a role in the long-term maintenance or worsening of CT reductions in (chronic) adult patients.

Some limitations should be considered. First, the cytokines IL-6 or TNF-α are the most thoroughly studied in AN, but other cytokines (e.g., IL-17A, IL-1β, IL-10) may also play an important role in inflammatory processes in AN and should be included in future studies as well as brain cell damage markers such as NF-L and GFAP. Furthermore, we only measured markers of peripheral inflammation and not central inflammatory markers, which would more accurately reflect neuroinflammation. Similarly, we have tested for associations with structural brain measures, but rapid inflammatory changes could impact neural function in ways that would change macroscopic brain structure. Second, our findings may be specific to young and non-chronic patients with AN. However, a sub-inflammatory state in adult chronic patients may also be related to deteriorating living conditions due to socioeconomic decline and increasing health burden rather than specifically to AN [63]. Multiple influencing factors on cytokine concentrations, such as smoking, age, psychiatric and somatic comorbidities and immunomodulating medication, were addressed in our inclusion criteria and additional sub-analyses. Furthermore, psychosocial stress, which we did not measure directly, may influence cytokine concentrations. Third, the relatively small number of patients with AN of the binge/purge subtype did not enable us to investigate the effect of AN subtype. Nonetheless, since our results did not change when excluding these participants, the impact of subtype seemed negligible in the current study. Finally, as all participants were all female and 98% identified as White, our findings cannot be generalized to all individuals with AN.

In conclusion, the present study provides no evidence for a link between measurable (sub-)inflammatory states (measured in peripheral blood) and the well-established widespread reductions of CT and gyrification in young patients with AN. Recent findings that brain regions with a higher density of energetically demanding cells have been found to be most affected by CT reduction [18] implicate nutritional status as an important factor in structural brain changes in acute AN. However, the present study cannot outrule neuroinflammation as a potential influence factor for structural brain changes. Future large-scale studies are needed to elucidate which other metabolic [18], hormonal [64] or cell-damaging processes [33] may also contribute to CT reduction in AN and should include further relevant parameters, such as MRI measures of neuroinflammation, measures of permeability of the blood-brain barrier or glymphatic measures. They should also explore differences between young individuals with a short duration of illness and adults with severe and enduring AN and include multiple longitudinal timepoints.

### Supplementary information

Supplementary Materials

## Data Availability

The datasets generated during and/or analyzed during the current study are available from the corresponding author on reasonable request.
