# Supplementary material for: Effect of serum concentrations of IL-6 and TNF-α on brain structure in anorexia nervosa: a combined cross-sectional and longitudinal study
Source: Neuropsychopharmacology. 2024 Mar 9;49(10):1509–17. doi: 10.1038/s41386-024-01836-z (PMC11319803; doi:10.1038/s41386-024-01836-z)
Supplement: Supplementary file 1 — Supplementary Materials [file 41386_2024_1836_MOESM1_ESM.docx]

**Supplementary Materials for “Effect of serum concentrations of IL-6 and TNF-α on brain structure in anorexia nervosa: a combined cross-sectional and longitudinal study”**

# Supplement 1: Methods

## **1.1 Participants**

The diagnostic procedures of the current study were identical to those in our previous cross-sectional study of cortical thickness (CT) in anorexia nervosa (AN) [1], as well as our previous longitudinal studies [2,3]. 79% of the participants were also included in our previous study [4]. Some parts of raw MRI and blood derived marker data included in the current manuscript have been used in manuscripts previously published by this group (Translational Developmental Neuroscience Section, Division of Psychological and Social Medicine and Developmental Neurosciences, Faculty of Medicine, TU Dresden, Dresden, Germany; PI Stefan Ehrlich, MD, PhD). The research questions, hypotheses, and analyses of the current manuscript are novel and unique and have not been published previously in any form.

Participants of all groups were excluded if they had a history of any of the following diagnoses: organic brain syndrome, dementia, schizophrenia, psychosis not otherwise specified, bipolar disorder, bulimia nervosa or binge-eating disorder. Further exclusion criteria for all participants were intelligence quotient (IQ) <85, antihistamine or antibiotic intake within two weeks prior to study participation, psychotropic medication intake within four weeks prior to the study apart from certain antidepressants (3 acAN-TP1, 1 acAN-TP2, 3 recAN were taking a selective serotonin reuptake inhibitor or a noradrenergic and specific serotonergic antidepressant), excessive smoking (>15 cigarettes per day), inflammatory, neurologic or metabolic illness, chronic medical or neurological illness that could affect appetite, eating behavior, or body weight (e.g., diabetes mellitus), clinically relevant anemia, pregnancy or breast feeding.

Comorbid psychiatric diagnoses were assessed in patients with AN by an expert clinician leading the treatment team and included examinations of the patients, discussions with the treatment staff and careful chart review (including consideration of medical and psychiatric history, physical examination, routine blood tests, urine analysis and several psychiatric screening instruments). Of the included acAN-T1 participants, 10/82 had one or more current psychiatric comorbidities (4/82 depressive disorder, 1/82 generalized anxiety disorder, 3/82 social phobia, 1/82 obsessive compulsive disorder, 2/82 tic disorder, 1/82 somatic symptom disorder, 1/82 adjustment disorder, 1/82 combined personality disorder, 1/82 suspected borderline personality disorder, 1/82 mental and behavioral disorders due to use of alcohol (harmful use), 1/82 mental and behavioral disorders due to use of cannabinoids (harmful use), 1/82 dyscalculia). Of the included recAN participants, 7/20 had one or more current or past psychiatric comorbidities (7/20 depressive disorder, 1/20 post traumatic stress disorder).

One or multiple physical illnesses with possible relevance for the research question (current (i.e. within two weeks before study participation) or recent (i.e. occurring within six weeks before study participation) infection or local inflammation; current or past diagnosis of neurodermitis, asthma, allergies or inflammatory disease) were reported by 13/82 acAN-TP1 participants (4/82 current or recent infection or local inflammation, 1/82 recent appendectomy, 3/82 neurodermitis, 1/82 asthma, 2/82 allergies, 1/82 epidermolysis bullosa simplex, 1/82 past mycobacterial infection, 1/82 past borreliosis in infancy). At acAN-TP2, 12/59 reported one or multiple potentially relevant physical illnesses (9/59 current or recent infection or local inflammation, 2/59 neurodermitis, 1/59 asthma, 1/59 allergies, 1/59 epidermolysis bullosa simplex, 1/59 past borreliosis). Of the recAN participants, 3/20 reported one or multiple potentially relevant physical illnesses (2/20 current or recent infection or local inflammation, 1/20 asthma, 1/20 allergies). Of the HC, 24/105 reported one or multiple potentially relevant physical illnesses (12/105 current or recent infection or local inflammation, 1/105 neurodermitis,3/105 asthma, 5/105 allergies, 1/105 past borreliosis, 1/105 past meningitis, 1/105 past autoimmune disease (unspecified)).

## **1.2 Leptin imputation**

Left-censored leptin concentrations below the lower limit of detection of the applied leptin assay (LOD=0.20ng/mL, 20/82 (24.4%) of acAN-TP1 participants in our sample had leptin values below LOD) were imputed using a quantile regression multiple imputation approach for left-censored missing data (QRILC). QRILC performs random draws from a truncated distribution with parameters estimated using quantile regression (derived from the distribution of existing leptin concentrations within detection range, please note that leptin values were log_e_-transformed and that no further covariates were introduced in the imputation model). QRILC was conducted in R with the help of package "imputeLCMD" [5]. A Gibbs sampler based approach [6] with n=100 iterative draws per value from the specified truncated distribution was then used to update the initialized values from QRILC and to ensure that the imputed leptin values were positive (on the original scale, i.e., >0) and below LOD.

## **1.3 Structural Image Acquisition**

High-resolution T1-weighted structural scans were acquired on a 3.0 T scanner (Magnetom Trio, Siemens, Erlangen, Germany) using a rapid acquisition gradient echo (MP-RAGE) sequence with the following parameters: 176 sagittal slices (1 mm thickness, no gap), T_R_=1900 ms; T_E_=2.26 ms; flip angle=9°; voxel size=1.0×1.0×1.0 mm, FoV=256 × 224 mm^2^, bandwidth of 200 Hz/pixel). 74% of the scans used for this study were already used for a previous publication [4]

## **1.4 Freesurfer Preprocessing**

Raw images were processed using standard FreeSurfer procedures (http://surfer.nmr.mgh.harvard.edu/; version 7.1.0) to achieve a sub-millimeter reconstruction of the cerebral cortex. The technical details of these procedures are described in prior publications [7–15]. In sum, images underwent motion correction, skull stripping, automated Talairach transformation, segmentation of the subcortical white matter and deep gray matter volumetric structures, intensity normalization, tessellation of the gray-white matter boundary, automated topology correction, and surface deformation following intensity gradients to optimally place the gray-white and gray matter-cerebrospinal fluid borders (white-matter and pial surfaces) at the location where the greatest shift in intensity defines the transition to the other tissue class. Next, to account for within-subject correlations present in the images collected from the longitudinal sample, sMRI images underwent further processing with the FreeSurfer longitudinal stream [16]. The longitudinal stream retraces the same steps as the cross-sectional preprocessing and produces a reconstruction of the cerebral cortex, but starts with the creation of an unbiased within-subject template using robust, inverse consistent registration [17]. Several processing steps , such as skull stripping, Talairach transforms, atlas registration as well as spherical surface maps and parcellations are then initialized with common information from the within-subject template, significantly increasing reliability and statistical power [16]. The surface obtained can be inflated and registered to a spherical atlas which is based on individual location of main sulci and gyri. This allows comparison of cortical geometry across subjects and parcellation of the cerebral cortex into units with respect to gyral and sulcal structure.

**1.5 Quality control procedures**

After automatic reconstruction of the cerebral cortex and subcortical regions with FreeSurfer, standardized quality control was performed by trained raters following standardized procedures established within the ENIGMA consortium (http://enigma.ini.usc.edu/). Initially, the Freesurfer QA Tools were used to verify that all steps in the FreeSurfer recon-all stream were executed and in the correct order and to create detailed snapshots of various volumes and surfaces. If artifacts that exert a significant influence on parcellation (mainly dura inclusions in the pial surface) were detected while inspecting the snapshots, the corresponding scan was excluded from the analysis. Subsequently, all thickness/surface data for the cortical regions of the Desikan-Killiany atlas [8] and subcortical regions were extracted. Scans for which at least three of these measures deviated by more than 2.698 standard deviations relative to the group mean were inspected more closely by a trained rater using Freeview.

## **1.6 Measurement of CT and lGI**

CT and lGI were computed at each vertex on the tessellated pial surface using standard Freesurfer procedures.

CT was defined as the closest distance from the gray-white boundary to the pial surface. To compute lGI, an outer surface is created, by first dilating and then eroding (morphological closing) the pial surface with a sphere of diameter 15mm, enough to avoid that the outer surface ‘falls’ into the main sulci [18]. lGI is then computed at >60000 vertices on each hemisphere of the outer surface and estimates the amount of cortex buried within the sulcal folds in the surrounding area. Specifically, it is computed as the ratio between the surface of a circular region (with radius 25mm) of interest on the outer surface, centered at each vertex, and the surface of the corresponding region of interest on the pial surface [19]. To allow intra subject comparisons, lGI are then projected from the outer to the pial surface such that each vertex of the pial surface (>160000 per hemisphere) will receive a weighted part of the lGI value for its contribution, weighting being inversely proportionate to the distance [19].

## **1.7 Statistical Analysis**

To model the predicting factors of cortical measures CT/lGI the following Linear-Mixed-Effects-Model was used:

$$CT,lGI=A+\Delta_{recAN}+\Delta_{acAN}+B_{acAN}\left( b_{t}-b_{TP2} \right)+D_{acAN}\left( d_{t}-d_{TP2} \right)+C age$$

where $A$ is the random intercept representing CT/lGI for the HC reference group at the mean age, age is the mean-subtracted age of the participant at the time of scan and $C$ is the rate of age-related changes (in mm/year).$\Delta$ terms represent group differences (always at the mean age), in particular $\Delta_{recAN}$ and $\Delta_{acAN}$ are the group differences between recAN vs. HC and acAN-TP1 vs. HC respectively. $b_{t}$ equals BMI-SDS at time $t$, so that the parameter $B_{acAN}$ encodes the rate of CT/lGI change as a function of the increase in BMI-SDS between baseline (acAN-TP1) and follow-up (acAN-TP2). $B_{acAN}$ indicates the speed of CT/lGI change relative to the BMI-SDS increase during weight restoration therapy (in mm/SDS-unit). Similarly, $d_{t}$ equals the plasma concentrations of the cytokine IL-6 or TNF-α at time $t$, so that the parameter $D_{acAN}$ encodes the rate of CT/lGI change as a function of the increase in IL-6 or TNF-α concentrations between baseline (acAN-TP1) and follow-up (acAN-TP2). When we applied this model to the analysis of volumes of subcortical regions, we also included estimated intracranial volume as a covariate. Furthermore, to control for possible effects of smoking and nonlinear age effects, we included age squared and the variables “current number of cigarettes per day” and “ever-smoking status” as covariates in a supplementary analysis (He et al., 2014):

$$CT,lGI=A+\Delta_{recAN}+\Delta_{acAN}+B_{acAN}\left( b_{t}-b_{TP2} \right)+D_{acAN}\left( d_{t}-d_{TP2} \right)+C age$$

$$+E age^{2}+F smoker+G cigarettes per day$$

To investigate relationships between cytokine levels and brain structure in each group, we used general linear models with cytokine levels and age as covariates:

$$CT,lGI=A+B cytokine+C age$$

where cytokine represents the log_e_-transformed serum concentrations of the cytokines IL-6 or TNF-α.

## **1.8 Supplemental analyses**

To assess the effect of comorbidities and subtypes on the relationships between cytokine concentrations and structural changes in AN we conducted the above-mentioned analyses by excluding participants from the AN groups that had (i) psychiatric or somatic comorbidities and (ii) were of the binge/purge subtype.

# Supplement 2: Results

## **2.1 Confirmatory analysis with age as a covariate**

Even when age was covaried for, the acAN-TP1, the recAN and the HC groups did not display any significant differences in serum IL-6 concentrations or serum TNF-α concentrations. (IL-6: F(2,203)=1.64, p=0.196; TNF-α: F(2,203)=1.59, p=0.207).

## **2.2 Confirmatory analysis with smoking as a covariate**

Even when smoking (ever-smoking status and current cigarettes per day) was covaried for, the acAN-TP1, the recAN and the HC groups did not display any significant differences in serum IL-6 concentrations or serum TNF-α concentrations (IL-6: F(2,202)=1.70, p=0.185; TNF-α: F(2,202)=2.65, p=0.073).

## **2.3 Confirmatory analysis using non-parametric tests**

Even when using-non parametric tests (Kruskal-Wallis followed by post-hoc Dunn-tests, to compare cross-sectionally the groups acAN-TP1, recAN and HC, and Wilcoxon signed rank test, to compare longitudinally measures in acAN-TP1 and acAN-TP2), the acAN-TP1, the recAN and the HC groups did not display any significant differences (after FDR correction across cytokine type, q<0.05) in serum IL-6 concentrations or serum TNF-α concentrations (IL-6: $X^{2}=4.92$, q=0.171; TNF-α: $X^{2}=6.31$, q=0.085).

## **2.4 Bayesian analyses**

To evaluate support for the null hypothesis, we employed version 0.9.2 of the BayesFactor package in R. Specifically, we computed Bayes factors for the comparisons: acAN-TP1 vs. HC (as independent samples) and acAN-TP1 vs. acAN-TP2 (as paired samples). The ttestBF function utilized conducts the "JZS" t-test as described by Rouder et al. (2009) [20]. Bayesian analyses yielded anecdotal evidence in favor of the null hypothesis for the contrast acAN-TP1 vs. HC (BF_10_ (IL-6)=0.702, BF_10_ (TNF-α)=0.445), and moderate evidence in favor of the null hypothesis for the contrast acAN-TP1 vs. acAN-TP2 (BF_10_ (IL-6)=0.208, BF_10_ (TNF-α)=0.183).

## **2.5 Confirmatory analysis excluding participants with comorbidities**

Even when excluding participants with psychiatric and somatic comorbidities, the acAN-TP1, the recAN and the HC groups did not display any significant differences in serum IL-6 concentrations or serum TNF-α concentrations. (IL-6: F(2,150)=1.53, p=0.220; TNF-α: F(2,150)=1.09, p=0.338). Similarly, even when excluding participants with comorbidities from the longitudinal sample of acAN patients, no differences in serum concentrations over the course of short-term weight rehabilitation were revealed (IL-6: t(39)=-0.78, p=0.442; TNF-α: t(39)=-0.70, p=0.488).

## **2.6 Confirmatory analysis excluding acAN and recAN participants of the binge/purge subtype**

Even when excluding acAN and recAN participants of the binge/purge subtype, the acAN-TP1, the recAN and the HC groups did not display any significant differences in serum IL-6 concentrations or serum TNF-α concentrations. (IL-6: F(2,185)=1.49, p=0.229; TNF-α: F(2,185)=1.16, p=0.315). Similarly, even when excluding participants of the binge/purge subtype from the longitudinal sample of acAN patients, no differences in serum concentrations over the course of short-term weight rehabilitation were revealed (IL-6: t(48)=-1.48, p=0.147; TNF-α: t(48)=-1.03, p=0.307).

## Figures


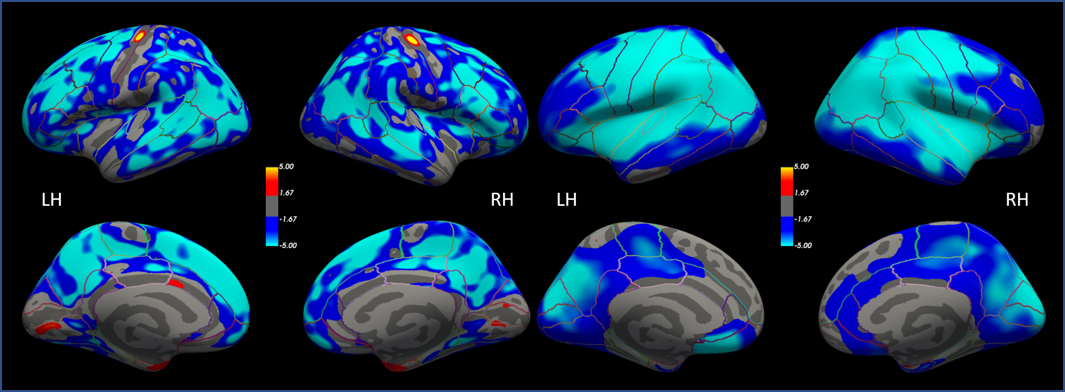


**Figure S1. Significant reductions in CT and IGI in acutely underweight patients scanned at the beginning of weight restoration treatment (acAN-TP1) compared to HC.**
**Left:** Reductions in CT. **Right:** Reductions in lGI. FDR-corrected statistical maps (q<0.05) plotted on the inflated surface of the standard average subject and displaying regions in which reductions in CT (top) and lGI (bottom) were observed in acAN-TP1 compared to HC. The color scale shows q-values expressed as −log_10_(q). Warm colors indicate increases in CT or lGI. LH, left hemisphere; RH, right hemisphere. Colored outlines correspond to anatomical labels of the Desikan-Killiany atlas [21].

**
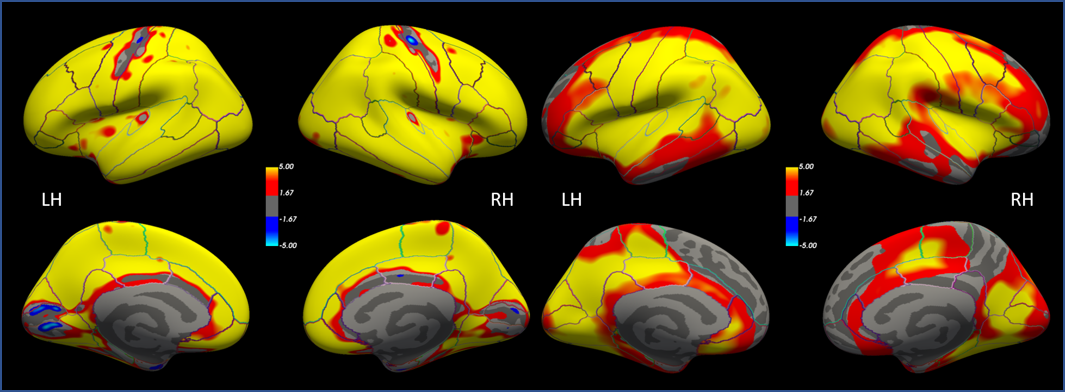
**

**Figure S2. Associations between longitudinal changes in cortical structure (acAN-TP2-acAN-TP1) and changes in BMI-SDS.**
**Left:** Associations with CT changes. **Right:** Associations with lGI. FDR-corrected statistical maps (q<0.05) plotted on the inflated surface of the standard average subject and displaying regions in which a treatment-related increase in BMI-SDS between acAN-TP1 and acAN-TP2 is associated with a significant change in CT or lGI. The color scale shows p values expressed as −log_10_(q). Warm colors indicate a positive correlation between changes in BMI-SDS concentrations and CT or lGI. LH, left hemisphere; RH, right hemisphere. Colored outlines correspond to anatomical labels of the Desikan-Killiany atlas [21].


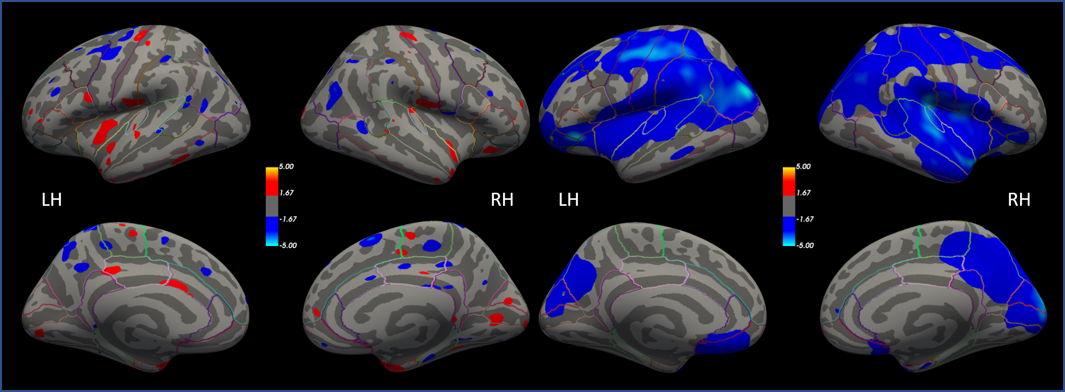


**Figure S3.** **Persisting lGI (but not CT) reduction in young patients with AN after short-term weight restoration treatment (acAN-TP2) compared to HC.**
**Left:** Reductions in CT. **Right:** Reductions in lGI. **Left:** Uncorrected statistical maps (p<0.05) plotted on the inflated surface of the standard average subject and displaying regions in which reductions in CT were observed in acAN-TP2 compared to HC. After FDR-correcting for multiple comparisons (q<0.05) these reductions were not significant. **Right:** FDR-corrected statistical maps (q<0.05) plotted on the inflated surface of the standard average subject and displaying regions in which reductions in lGI were observed in acAN-TP2 compared to HC. The color scale shows p values expressed as −log_10_(q). Warm colors indicate increases in CT or lGI. LH, left hemisphere; RH, right hemisphere. Colored outlines correspond to anatomical labels of the Desikan-Killiany atlas [21].


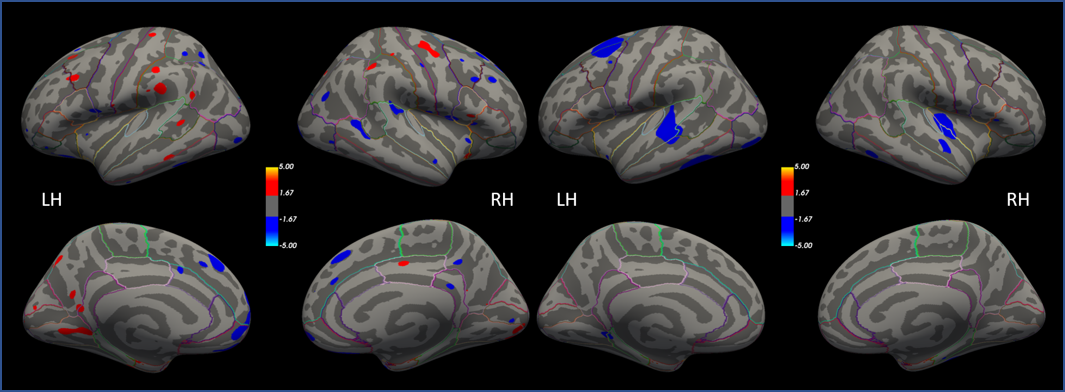


**Figure S4. No significant associations between serum concentrations of the IL-6 cytokine and cortical structure in acAN-TP1.**
**Left:** Associations with CT. **Right:** Associations with lGI. Uncorrected statistical maps (p<0.05) plotted on the inflated surface of the standard average subject and displaying regions in which linear association between CT or lGI and IL-6 concentrations were significant in acAN-TP1. The color scale shows p values expressed as −log_10_(p). Warm colors indicate a positive correlation between IL-6 concentrations and CT or lGI. After FDR-correcting for multiple comparisons (q<0.05) across each hemisphere and Bonferroni correcting across cytokine type, these associations were not significant. LH, left hemisphere; RH, right hemisphere. Colored outlines correspond to anatomical labels of the Desikan-Killiany atlas [21].


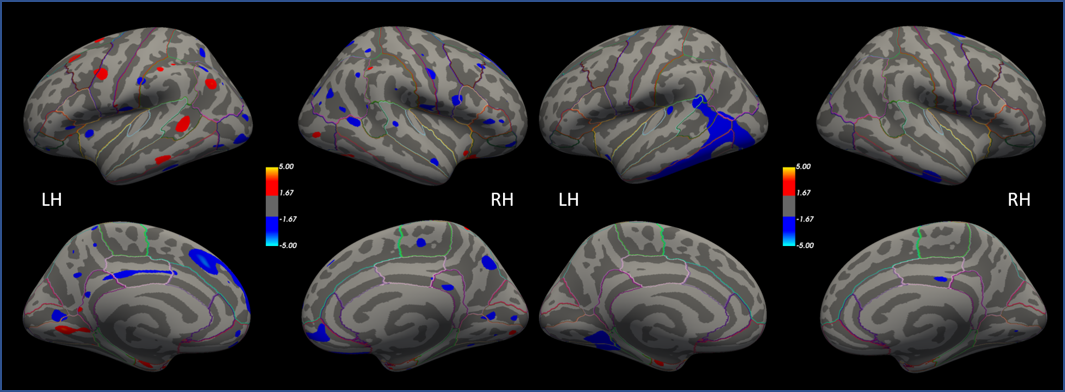


**Figure S5. No significant associations between serum concentrations of the IL-6 cytokine and cortical structure in acAN-TP2.**
**Left:** Associations with CT. **Right:** Associations with lGI. Uncorrected statistical maps (p<0.05) plotted on the inflated surface of the standard average subject and displaying regions in which linear association between CT or lGI and IL-6 concentrations were significant in acAN-TP2. The color scale shows p values expressed as −log_10_(p). Warm colors indicate a positive correlation between IL-6 concentrations and CT or lGI. After FDR-correcting for multiple comparisons (q<0.05) across each hemisphere and Bonferroni correcting across cytokine type, these associations were not significant. LH, left hemisphere; RH, right hemisphere. Colored outlines correspond to anatomical labels of the Desikan-Killiany atlas [21].


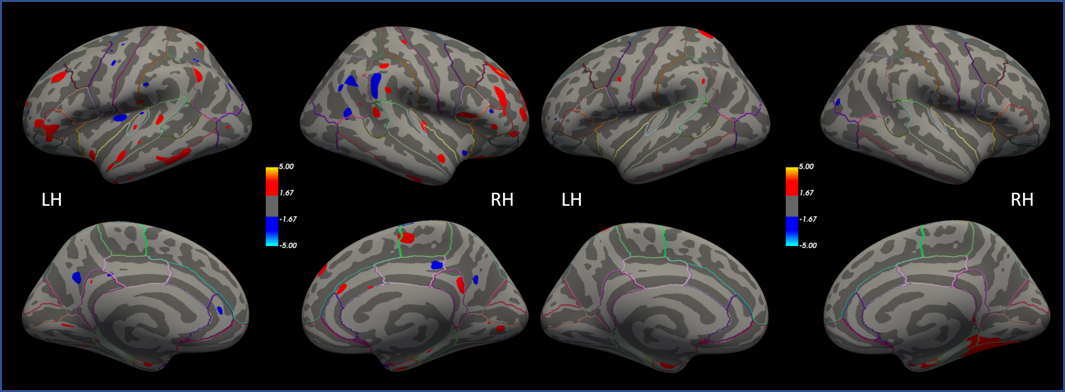


**Figure S6. No significant associations between serum concentrations of the IL-6 cytokine and cortical structure in recAN.**
**Left:** Associations with CT. **Right:** Associations with lGI. Uncorrected statistical maps (p<0.05) plotted on the inflated surface of the standard average subject and displaying regions in which linear association between CT or lGI and IL-6 concentrations were significant in recAN. The color scale shows p values expressed as −log_10_(p). Warm colors indicate a positive correlation between IL-6 concentrations and CT or lGI. After FDR-correcting for multiple comparisons (q<0.05) across each hemisphere and Bonferroni correcting across cytokine type, these associations were not significant. LH, left hemisphere; RH, right hemisphere. Colored outlines correspond to anatomical labels of the Desikan-Killiany atlas [21].


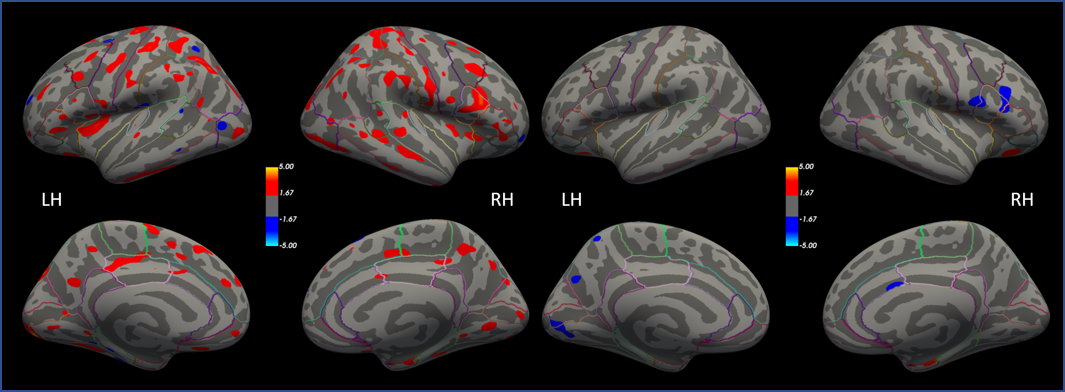


**Figure S7. No significant associations between serum concentrations of the IL-6 cytokine and cortical structure in HC.**
**Left:** Associations with CT. **Right:** Associations with lGI. Uncorrected statistical maps (p<0.05) plotted on the inflated surface of the standard average subject and displaying regions in which linear association between CT or lGI and IL-6 concentrations were significant in HC. The color scale shows p values expressed as −log_10_(p). Warm colors indicate a positive correlation between IL-6 concentrations and CT or lGI. After FDR-correcting for multiple comparisons (q<0.05) across each hemisphere and Bonferroni correcting across cytokine type, these associations were not significant. LH, left hemisphere; RH, right hemisphere. Colored outlines correspond to anatomical labels of the Desikan-Killiany atlas [21].


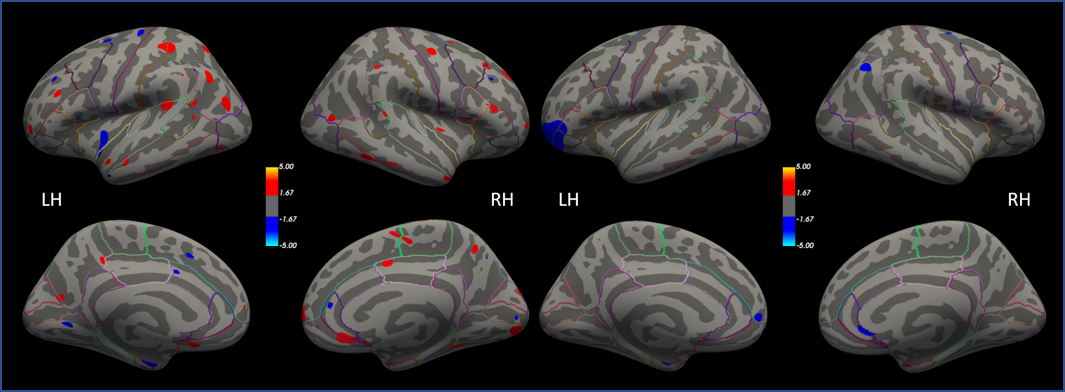


**Figure S8. No significant associations between serum concentrations of TNF-α and cortical structure in acAN-TP1.**
**Left:** Associations with CT. **Right:** Associations with lGI. Uncorrected statistical maps (p<0.05) plotted on the inflated surface of the standard average subject and displaying regions in which linear association between CT or lGI and TNF-α concentrations were significant in acAN-TP1. The color scale shows p values expressed as −log_10_(p). Warm colors indicate a positive correlation between TNF-α concentrations and CT or lGI. After FDR-correcting for multiple comparisons (q<0.05) across each hemisphere and Bonferroni correcting across cytokine type, these associations were not significant. LH, left hemisphere; RH, right hemisphere. Colored outlines correspond to anatomical labels of the Desikan-Killiany atlas [21].


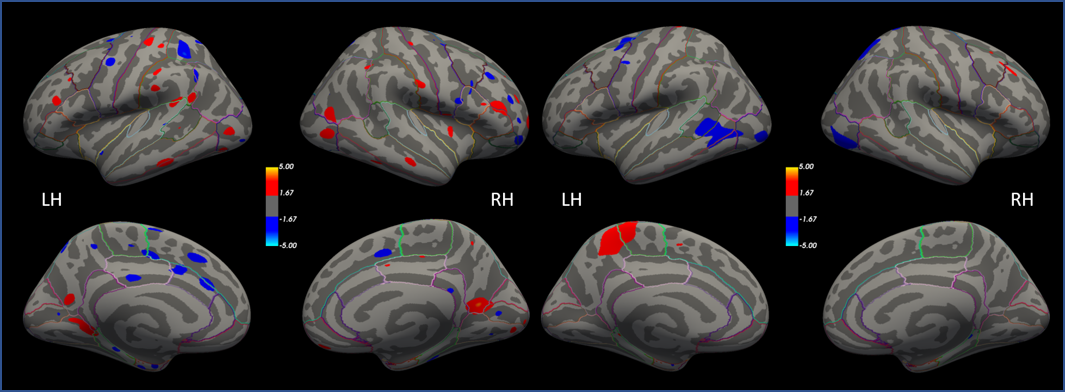


**Figure S9. No significant associations between serum concentrations of TNF-α and cortical structure in acAN-TP2.**
**Left:** Associations with CT. **Right:** Associations with lGI. Uncorrected statistical maps (p<0.05) plotted on the inflated surface of the standard average subject and displaying regions in which linear association between CT or lGI and TNF-α concentrations were significant in acAN-TP2. The color scale shows p values expressed as −log_10_(p). Warm colors indicate a positive correlation between TNF-α concentrations and CT or lGI. After FDR-correcting for multiple comparisons (q<0.05) across each hemisphere and Bonferroni correcting across cytokine type, these associations were not significant. LH, left hemisphere; RH, right hemisphere. Colored outlines correspond to anatomical labels of the Desikan-Killiany atlas [21].


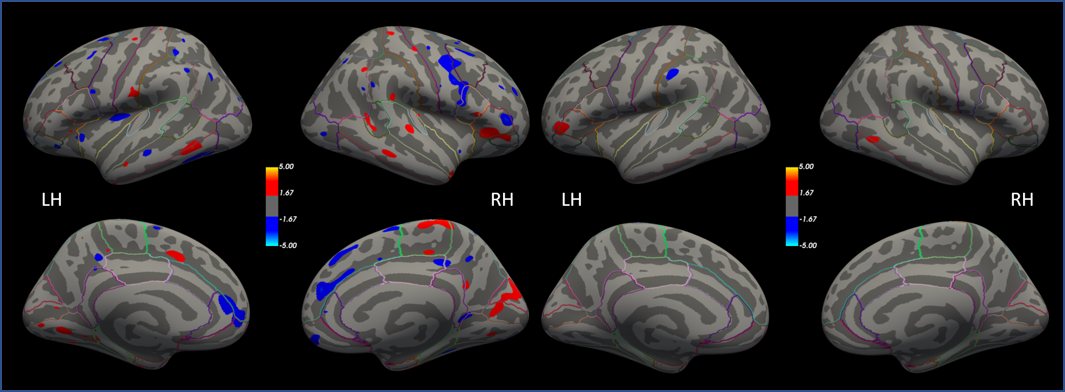


**Figure S10. No significant associations between serum concentrations of TNF-α and cortical structure in recAN.**
**Left:** Associations with CT. **Right:** Associations with lGI. Uncorrected statistical maps (p<0.05) plotted on the inflated surface of the standard average subject and displaying regions in which linear association between CT or lGI and TNF-α concentrations were significant in recAN. The color scale shows p values expressed as −log_10_(p). Warm colors indicate a positive correlation between TNF-α concentrations and CT or lGI. After FDR-correcting for multiple comparisons (q<0.05) across each hemisphere and Bonferroni correcting across cytokine type, these associations were not significant. LH, left hemisphere; RH, right hemisphere. Colored outlines correspond to anatomical labels of the Desikan-Killiany atlas [21].


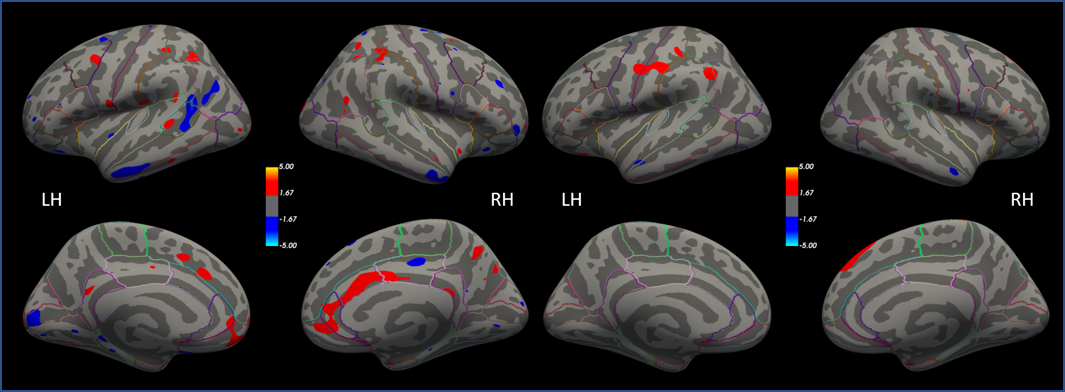


**Figure S11. No significant associations between serum concentrations of TNF-α and cortical structure in HC.**
**Left:** Associations with CT. **Right:** Associations with lGI. Uncorrected statistical maps (p<0.05) plotted on the inflated surface of the standard average subject and displaying regions in which linear association between CT or lGI and TNF-α concentrations were significant in HC. The color scale shows p values expressed as −log_10_(p). Warm colors indicate a positive correlation between TNF-α concentrations and CT or lGI. After FDR-correcting for multiple comparisons (q<0.05) across each hemisphere and Bonferroni correcting across cytokine type, these associations were not significant. LH, left hemisphere; RH, right hemisphere. Colored outlines correspond to anatomical labels of the Desikan-Killiany atlas [21].


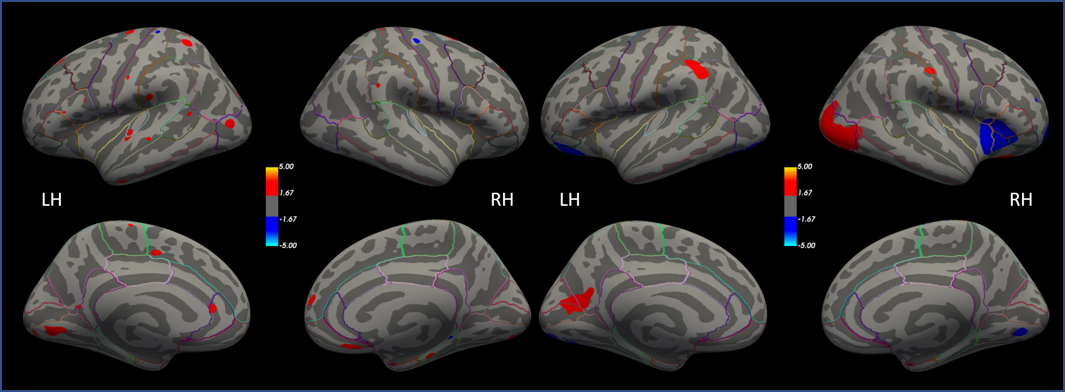


**Figure S12. No significant associations between longitudinal changes in cortical structure (acAN-TP2-acAN-TP1) and changes in serum concentrations of the IL-6 cytokine after excluding participants in the AN groups with comorbidities.**
**Left:** Associations with CT changes. **Right:** Associations with lGI changes. Uncorrected statistical maps (p<0.05) plotted on the inflated surface of the standard average subject and displaying regions in which differences in CT or lGI between acAN-TP2 and acAN-TP1 were associated with changes in IL-6. The color scale shows p values expressed as −log_10_(p). Warm colors indicate a positive correlation between changes in IL-6 concentrations and CT or lGI. After FDR-correcting for multiple comparisons (q<0.05) across each hemisphere and Bonferroni correcting across cytokine type, these associations were not significant. LH, left hemisphere; RH, right hemisphere. Colored outlines correspond to anatomical labels of the Desikan-Killiany atlas [21].


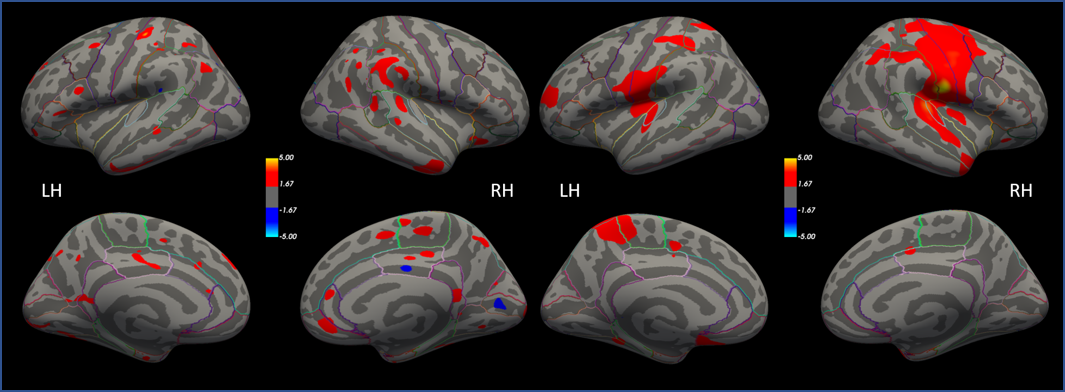


**Figure S13. No significant associations between longitudinal changes in cortical structure (acAN-TP2-acAN-TP1) and changes in serum concentrations of TNF-α after excluding participants in the AN groups with comorbidities.**
**Left:** Associations with CT changes. **Right:** Associations with lGI changes. Uncorrected statistical maps (p<0.05) plotted on the inflated surface of the standard average subject and displaying regions in which differences in CT or lGI between acAN-TP2 and acAN-TP1 were associated with changes in TNF-α. The color scale shows p values expressed as −log_10_(p). Warm colors indicate a positive correlation between changes in TNF-α concentrations and CT or lGI. After FDR-correcting for multiple comparisons (q<0.05) across each hemisphere and Bonferroni correcting across cytokine type, these associations were not significant. LH, left hemisphere; RH, right hemisphere. Colored outlines correspond to anatomical labels of the Desikan-Killiany atlas [21].


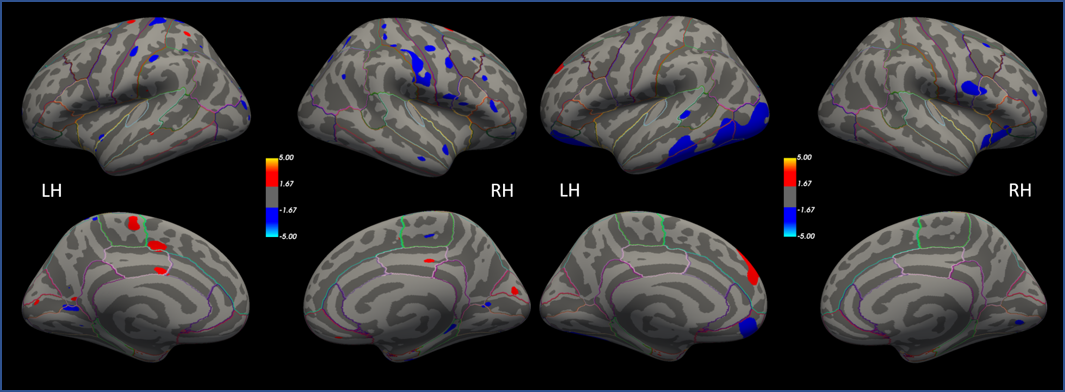


**Figure S14. No significant associations between longitudinal changes in cortical structure (acAN-TP2-acAN-TP1) and changes in serum concentrations of the IL-6 cytokine after excluding participants with the AN binge/purge subtype.**
**Left:** Associations with CT changes. **Right:** Associations with lGI changes. Uncorrected statistical maps (p<0.05) plotted on the inflated surface of the standard average subject and displaying regions in which differences in CT or lGI between acAN-TP2 and acAN-TP1 were associated with changes in IL-6. The color scale shows p values expressed as −log_10_(p). Warm colors indicate a positive correlation between changes in IL-6 concentrations and CT or lGI. After FDR-correcting for multiple comparisons (q<0.05) across each hemisphere and Bonferroni correcting across cytokine type, these associations were not significant. LH, left hemisphere; RH, right hemisphere. Colored outlines correspond to anatomical labels of the Desikan-Killiany atlas [21].

**
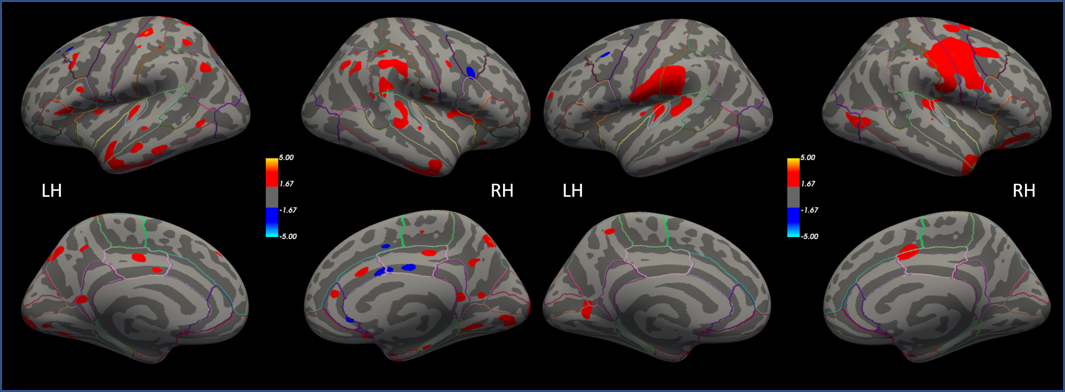
Figure S15. No significant associations between longitudinal changes in cortical structure (acAN-TP2-acAN-TP1) and changes in serum concentrations of TNF-α after excluding participants with the AN binge/purge subtype.**
**Left:** Associations with CT changes. **Right:** Associations with lGI. Uncorrected statistical maps (p<0.05) plotted on the inflated surface of the standard average subject and displaying regions in which differences in CT or lGI between acAN-TP2 and acAN-TP1 were associated with changes in TNF-α. The color scale shows p values expressed as −log_10_(p). Warm colors indicate a positive correlation between changes in TNF-α concentrations and CT or lGI. After FDR-correcting for multiple comparisons (q<0.05) across each hemisphere and Bonferroni correcting across cytokine type, these associations were not significant. LH, left hemisphere; RH, right hemisphere. Colored outlines correspond to anatomical labels of the Desikan-Killiany atlas [21].


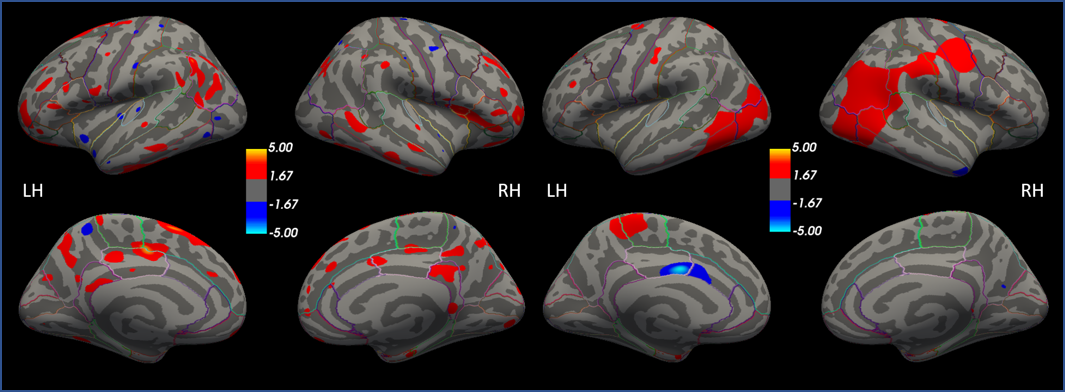


**Figure S16. No significant associations between age squared and cortical structure in our study sample.**
**Left:** Associations with CT. **Right:** Associations with lGI. Uncorrected statistical maps (p<0.05) plotted on the inflated surface of the standard average subject and displaying regions in which CT or lGI were associated with age^2^. The color scale shows p values expressed as −log_10_(p). Warm colors indicate a positive correlation between age^2^ and CT or lGI. After FDR-correcting for multiple comparisons (q<0.05) these associations were not significant. LH, left hemisphere; RH, right hemisphere. Colored outlines correspond to anatomical labels of the Desikan-Killiany atlas [21].


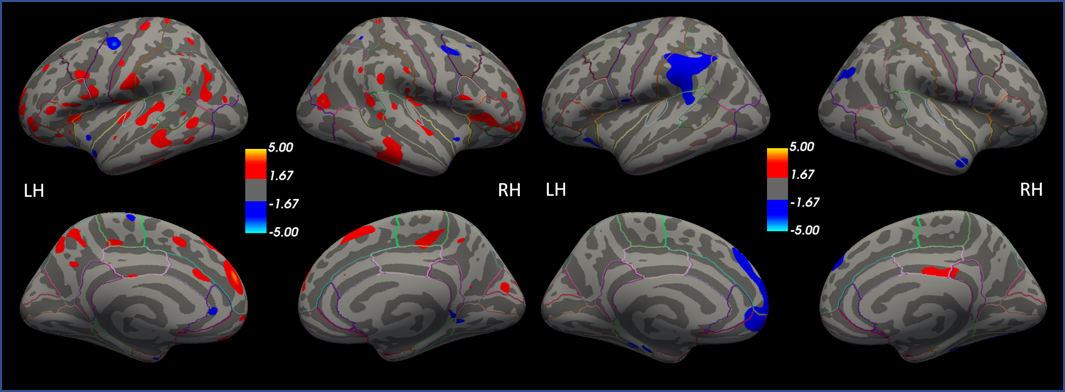


**Figure S17. No significant associations between ever-smoking status and cortical structure in our study sample.**
**Left:** Associations with CT. **Right:** Associations with lGI. Uncorrected statistical maps (p<0.05) plotted on the inflated surface of the standard average subject and displaying regions in which CT or lGI were associated with smoker status. The color scale shows p values expressed as −log_10_(p). Warm colors indicate increased CT or lGI in smokers. After FDR-correcting for multiple comparisons (q<0.05) these associations were not significant. LH, left hemisphere; RH, right hemisphere. Colored outlines correspond to anatomical labels of the Desikan-Killiany atlas [21].


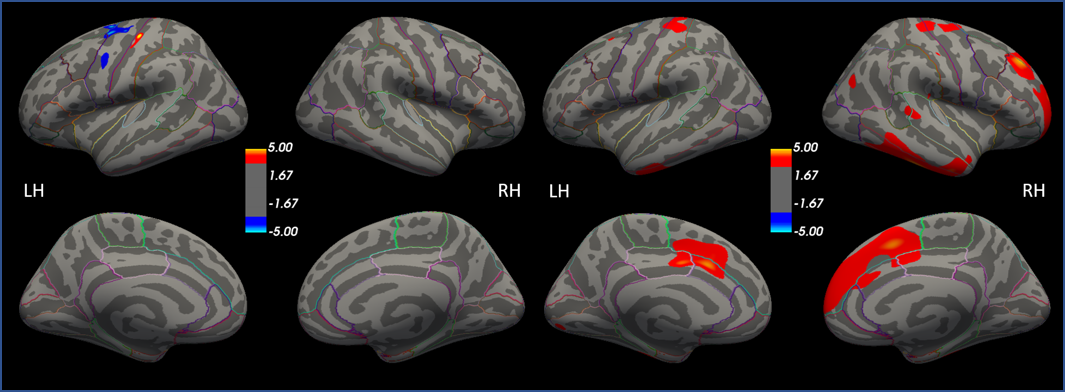


**Figure S18. Significant associations between cigarettes smoked per day and CT, but not between cigarettes smoked per day and lGI.**
**Left:** Associations with CT. FDR-corrected statistical maps (q<0.05) displaying regions in which CT was associated with “current number of cigarettes per day”. **Right:** Associations with lGI. Uncorrected statistical maps (p<0.05). After FDR-correcting for multiple comparisons (q<0.05) these associations were not significant. Statistical maps were plotted on the inflated surface of the standard average subject. The color scale shows p values expressed as −log_10_(p). Warm colors indicate a positive association between CT or lGI and “current number of cigarettes per day”. LH, left hemisphere; RH, right hemisphere. Colored outlines correspond to anatomical labels of the Desikan-Killiany atlas [21].


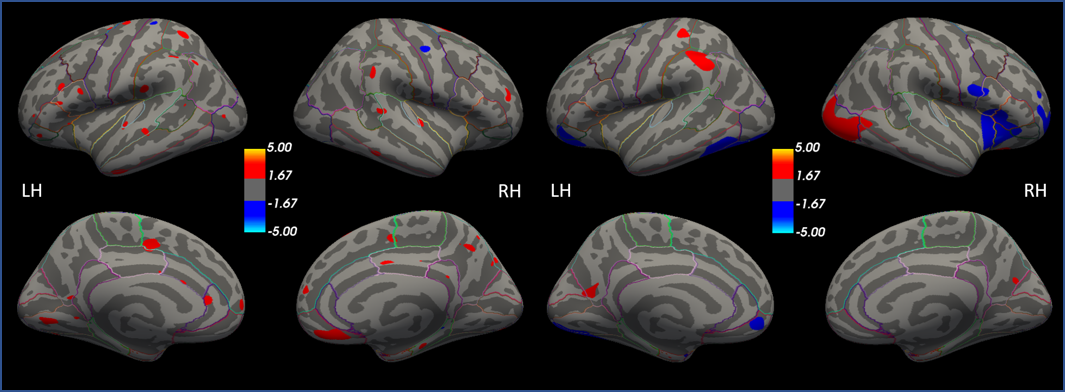


**Figure S19. No significant associations between longitudinal changes in cortical structure (acAN-TP2-acAN-TP1) and changes in serum concentrations of the IL-6 cytokine after accounting for effects of smoking and nonlinear effects of age.**
**Left:** Associations with CT changes. **Right:** Associations with lGI changes. We controlled for possible effects of smoking and nonlinear age effects by including age squared and the variables “current number of cigarettes per day” and “ever-smoking status” as covariates (Figures S17-S18). Uncorrected statistical maps (p<0.05) plotted on the inflated surface of the standard average subject and displaying regions in which differences in CT or lGI between acAN-TP2 and acAN-TP1 were associated with changes in IL-6. The color scale shows p values expressed as −log_10_(p). Warm colors indicate a positive correlation between changes in IL-6 concentrations and CT or lGI. After FDR-correcting for multiple comparisons (q<0.05) across each hemisphere and Bonferroni correcting across cytokine type, these associations were not significant. LH, left hemisphere; RH, right hemisphere. Colored outlines correspond to anatomical labels of the Desikan-Killiany atlas [21].


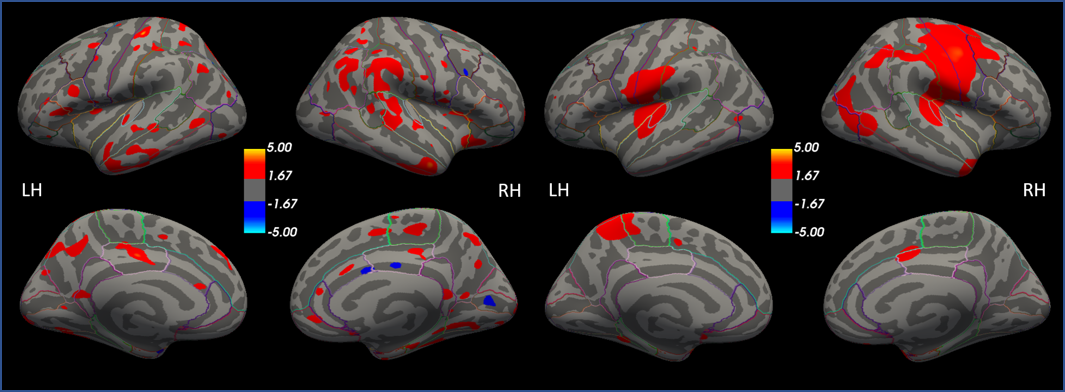
**Figure S20. No significant associations between longitudinal changes in cortical structure (acAN-TP2-acAN-TP1) and changes in serum concentrations of TNF-α after accounting for effects of smoking and nonlinear effects of age.**
**Left:** Associations with CT changes. **Right:** Associations with lGI changes. We controlled for possible effects of smoking and nonlinear age effects by including age squared and the variables “current number of cigarettes per day” and “ever-smoking status” as covariates (Figure S17-S18). Uncorrected statistical maps (p<0.05) plotted on the inflated surface of the standard average subject and displaying regions in which differences in CT or lGI between acAN-TP2 and acAN-TP1 were associated with changes in TNF-α. The color scale shows p values expressed as −log_10_(p). Warm colors indicate a positive correlation between changes in TNF-α concentrations and CT or lGI changes. After FDR-correcting for multiple comparisons (q<0.05) across each hemisphere and Bonferroni correcting across cytokine type, these associations were not significant. LH, left hemisphere; RH, right hemisphere. Colored outlines correspond to anatomical labels of the Desikan-Killiany atlas [21].

## Tables

**Table S1. Correlations of cytokines with clinical variables**

|  |  | EDI-2 total score | BDI-II total score | SCL-90-R anxiety scale | BMI-SDS | Leptin |
| --- | --- | --- | --- | --- | --- | --- |
| AN-TP1 | IL-6 | *r_s_*=-0.04  *p*=0.698  *q*=0.776  N=81 | *r_s_*=-0.08  *p*=0.498  *q*=0.623  N=82 | *r_s_*=-0.14  *p*=0.219  *q*=0.366  N=82 | *r_s_*=0.01  *p=*0.963  *q*=0.963  N=82 | *r_s_*=0.13  *p=*0.264  *q*=0.377  N=82 |
|  | TNF-α | *r_s_*=-0.15  *p=*0.179  *q*=0.366  N=81 | *r_s_*=-0.21  *p=*0.054  *q*=0.269  N=82 | *r_s_*=-0.27  *p*=0.014  *q*=0.140  N=82 | *r_s_*=0.16  *p=*0.165  *q*=0.366  N=82 | *r_s_*=0.14  *p=*0.197  *q*=0.366  N=81 |
| AN-TP2 | IL-6 | *r_s_*=-0.12  *p*=0.386  *q*=0.451  N=58 | *r_s_*=-0.12  *p*=0.355  *q*=0.451  N=59 | *r_s_*=-0.11  *p*=0.406  *q*=0.451  N=59 | *r_s_*=0.10  *p*=0.452  *q*=0.452  N=59 | *r_s_*=0.17  *p*=0.190  *q*=0.451  N=59 |
|  | TNF-α | *r_s_*=-0.12  *p*=0.355  *q*=0.451  N=58 | *r_s_*=-0.23  *p*=0.083  *q*=0.417  N=59 | *r_s_*=-0.23  *p*=0.083  *q*=0.417  N=59 | *r_s_*=0.17  *p*=0.197  *q*=0.451  N=59 | *r_s_*=0.12  *p*=0.385  *q*=0.451  N=59 |
| recAN | IL-6 | *r_s_*=0.00  *p*=0.989  *q*=0.989  N=19 | *r_s_*=-0.19  *p*=0.449  *q*=0.919  N=19 | *r_s_*=-0.10  *p*=0.686  *q*=0.972  N=20 | *r_s_*=-0.04  *p*=0.875  *q*=0.972  N=20 | *r_s_*=0.05  *p*=0.845  *q*=0.972  N=20 |
|  | TNF-α | *r_s_*=-0.22  *p*=0.377  *q*=0.919  N=19 | *r_s_*=-0.23  *p*=0.342  *q*=0.919  N=19 | *r_s_*=0.14  *p*=0.551  *q*=0.919  N=20 | *r_s_*=-0.14  *p*=0.544  *q*=0.919  N=20 | *r_s_*=-0.31  *p*=0.181  *q*=0.919  N=20 |
| HC | IL-6 | *r_s_*=-0.03  *p*=0.749  *q*=0.832  N=102 | *r_s_*=-0.12  *p*=0.233  *q*=0.816  N=104 | *r_s_*=0.04  *p*=0.691  *q*=0.832  N=103 | *r_s_*=0.08  *p*=0.431  *q*=0.832  N=105 | *r_s_*=0.01  *p*=0.959  *q*=0.959  N=104 |
|  | TNF-α | *r_s_*=-0.12  *p*=0.232  *q*=0.816  N=102 | *r_s_*=-0.12  *p*=0.245  *q*=0.816  N=104 | *r_s_*=0.04  *p*=0.687  *q*=0.832  N=103 | *r_s_*=-0.04  *p*=0.722  *q*=0.832  N=105 | *r_s_*=0.04  *p*=0.722  *q*=0.832  N=104 |

Spearman correlation coefficients *r_s_* between cytokine concentrations and clinical variables. For each group separately, we report uncorrected p-values and p-values adjusted for multiple comparisons (i.e., q, 10 tests per group) using the False Discovery Rate (FDR) method of Benjamini and Hochberg [22]. Asterisks denote a significant group difference after multiple comparison correction: *=*q*<0.05, **=*q*<0.01, ***=*q*<0.001. Abbreviations: acAN-TP1, acute anorexia nervosa participants at time point 1 (admission); acAN-TP2, acute anorexia nervosa participants at time point 2 (after short-term weight restoration); BDI-II, Beck Depression Inventory; EDI-2, Eating Disorder Inventory-2; HC, healthy control participants; recAN, long-term weight-recovered anorexia nervosa participants.

**Table S2. Correlations of neurofilament light with cytokines in the study sample of Hellerhoff et al.** [23]

|  |  | IL-6 | TNF-α |
| --- | --- | --- | --- |
| Neurofilament light | acAN-TP1  (N=54) | *r_s_*=0.05  *p*=0.717  *q*=0.838 | *r_s_*=-0.03  *p*=0.838  *q*=0.838 |
|  | acAN-TP2  (N=53) | *r_s_*=0.20  *p*=0.158  *q*=0.316 | *r_s_*=-0.11  *p*=0.432  *q*=0.432 |
|  | HC  (N=99) | *r_s_*=0.04  *p*=0.660  *q*=0.717 | *r_s_*=-0.04  *p*=0.717  *q*=0.717 |

Spearman correlation coefficients *r_s_* between neurofilament light and cytokine concentrations. For each group separately, we report raw *p-*values and *p-*values adjusted for multiple comparisons (i.e., q, 2 tests per group) using the False Discovery Rate (FDR) method of Benjamini and Hochberg [22]. Asterisks denote a significant group difference after multiple comparison correction: *=*q*<0.05, **=*q*<0.01, ***=*q*<0.001. Abbreviations: acAN-TP1, acute anorexia nervosa participants at time point 1 (admission); acAN-TP2, acute anorexia nervosa participants at time point 2 (after short-term weight restoration); HC, healthy control participants.

**Table S3. No significant associations between longitudinal changes in volumes of subcortical structures (acAN-TP2-acAN-TP1) and changes in serum concentrations of the IL-6 and TNF-α cytokines.**

| Region |  | Sample | | | |  | Statistical inference | | |  |
| --- | --- | --- | --- | --- | --- | --- | --- | --- | --- | --- |
|  |  | acAN-TP1 | acAN-TP2 | recAN | HC |  | HC – acAN-TP1 | ΔBMI-SDS | ΔTNF-α | ΔIL-6 |
|  |  |  |  |  |  |  | F / p / dof | F / p / dof | F / p / dof | F / p / dof |
| Accumbens [cm^2^] |  | 5.20(0.91) | 5.41(0.83) | 5.57(1.17) | 5.53(1.06) |  | 20.84 | 41.24 | 0.02 | 5.14 |
|  |  |  |  |  |  |  | 0.0000 | 0.0000 | 0.8792 | 0.0270 |
|  |  |  |  |  |  |  | 204.92 | 59.53 | 60.55 | 60.20 |
| Amygdala [cm^3^] |  | 1.68(0.18) | 1.72(0.17) | 1.68(0.19) | 1.74(0.18) |  | 19.34 | 22.66 | 0.79 | 0.63 |
|  |  |  |  |  |  |  | 0.0000 | 0.0000 | 0.3773 | 0.4295 |
|  |  |  |  |  |  |  | 204.92 | 59.53 | 60.55 | 60.62 |
| Caudate [cm^3^] |  | 3.86(0.45) | 4.04(0.44) | 3.96(0.45) | 4.01(0.46) |  | 15.94 | 180.00 | 0.01 | 0.32 |
|  |  |  |  |  |  |  | 0.0001 | 0.0000 | 0.9247 | 0.5712 |
|  |  |  |  |  |  |  | 203.48 | 60.17 | 58.75 | 58.75 |
| Cerebellum [cm^3^] |  | 58.60(5.36) | 60.71(5.07) | 61.28(4.81) | 60.30(5.30) |  | 11.93 | 166.96 | 0.33 | 0.12 |
|  |  |  |  |  |  |  | 0.0007 | 0.0000 | 0.5654 | 0.7294 |
|  |  |  |  |  |  |  | 203.47 | 60.18 | 58.74 | 58.73 |
| Hippocampus [cm^3^] |  | 4.22(0.41) | 4.41(0.40) | 4.48(0.44) | 4.38(0.40) |  | 21.59 | 124.50 | 0.18 | 1.37 |
|  |  |  |  |  |  |  | 0.0000 | 0.0000 | 0.6759 | 0.2465 |
|  |  |  |  |  |  |  | 205.32 | 59.55 | 61.06 | 61.07 |
| Pallidum [cm^3^] |  | 2.03(0.21) | 2.02(0.21) | 1.97(0.22) | 2.06(0.21) |  | 3.66 | 18.64 | 0.13 | 0.12 |
|  |  |  |  |  |  |  | 0.0570 | 0.0001 | 0.7167 | 0.7304 |
|  |  |  |  |  |  |  | 204.11 | 59.68 | 59.53 | 59.54 |
| Putamen [cm^3^] |  | 5.30(0.55) | 5.45(0.54) | 5.21(0.56) | 5.42(0.55) |  | 4.74 | 68.82 | 0.00 | 3.80 |
|  |  |  |  |  |  |  | 0.0306 | 0.0000 | 0.9693 | 0.0561 |
|  |  |  |  |  |  |  | 203.92 | 59.77 | 59.29 | 59.14 |
| Thalamus [cm^3^] |  | 7.93(0.76) | 8.36(0.76) | 8.34(0.82) | 8.29(0.78) |  | 37.44 | 169.21 | 0.04 | 0.67 |
|  |  |  |  |  |  |  | 0.0000 | 0.0000 | 0.8355 | 0.4146 |
|  |  |  |  |  |  |  | 206.18 | 59.70 | 62.19 | 62.17 |

Mean Volume estimates for subcortical gray matter nuclei (mean(SD) cm^3^, averaged across both hemispheres; for the Nucleus Accumbens surface in cm^2^ instead of volume is given) as produced by the Freesurfer segmentation and for each group (acAN-TP1, acAN-TP2, recAN, HC). Statistical inference was performed based on the LME model using changes in BMI-SDS and the log_e_-transformed serum concentrations of the cytokines IL-6 or TNF-α to predict longitudinal changes (between acAN-TP1 and acAN-TP2), while covarying for age and intracranial volume. We reproduced our results of reduced volumes in acAN-TP1 compared to HC, and longitudinal increase (between acAN-TP1 and acAN-TP2) following weight restoration associated with BMI-SDS [4]. No differences between HC and acAN-TP2 or recAN were significant. After correcting for multiple comparisons across regions (FDR), no effect of IL-6 or TNF-α increase on the volumetric changes was significant (q>0.2). To facilitate comparison with future studies, uncorrected p-values are reported. At this more lenient threshold, an increase in the IL-6 levels was associated with and increase in the Accumbens surface.

## References

1. King JA, Geisler D, Ritschel F, Boehm I, Seidel M, Roschinski B, et al. Global cortical thinning in acute anorexia nervosa normalizes following long-term weight restoration. Biological Psychiatry. 2015;77:624–632.

2. Bernardoni F, King JA, Geisler D, Stein E, Jaite C, Nätsch D, et al. Weight restoration therapy rapidly reverses cortical thinning in anorexia nervosa: A longitudinal study. NeuroImage. 2016;130:214–222.

3. Bernardoni F, King JA, Geisler D, Birkenstock J, Tam FI, Weidner K, et al. Nutritional Status Affects Cortical Folding: Lessons Learned From Anorexia Nervosa. Biological Psychiatry. 2018;84:692–701.

4. Bahnsen K, Bernardoni F, King JA, Geisler D, Weidner K, Roessner V, et al. Dynamic Structural Brain Changes in Anorexia Nervosa: A Replication Study, Mega-analysis, and Virtual Histology Approach. Journal of the American Academy of Child & Adolescent Psychiatry. 2022:S0890856722001836.

5. Lazar C. imputeLCMD: A collection of methods for left-censored missing data imputation. R package version 2.0. 2015.

6. Wei R, Wang J, Jia E, Chen T, Ni Y, Jia W. GSimp: A Gibbs sampler based left-censored missing value imputation approach for metabolomics studies. PLoS Comput Biol. 2018;14:e1005973.

7. Dale AM, Fischl B, Sereno MI. Cortical surface-based analysis. I. Segmentation and surface reconstruction. Neuroimage. 1999;9:179–194.

8. Desikan RS, Ségonne F, Fischl B, Quinn BT, Dickerson BC, Blacker D, et al. An automated labeling system for subdividing the human cerebral cortex on MRI scans into gyral based regions of interest. Neuroimage. 2006;31:968–980.

9. Fischl B, Salat DH, van der Kouwe AJW, Makris N, Ségonne F, Quinn BT, et al. Sequence-independent segmentation of magnetic resonance images. Neuroimage. 2004;23 Suppl 1:S69-84.

10. Fischl B, van der Kouwe A, Destrieux C, Halgren E, Ségonne F, Salat DH, et al. Automatically parcellating the human cerebral cortex. Cereb Cortex. 2004;14:11–22.

11. Fischl B, Salat DH, Busa E, Albert M, Dieterich M, Haselgrove C, et al. Whole brain segmentation: automated labeling of neuroanatomical structures in the human brain. Neuron. 2002;33:341–355.

12. Fischl B, Sereno MI, Dale AM. Cortical surface-based analysis. II: Inflation, flattening, and a surface-based coordinate system. Neuroimage. 1999;9:195–207.

13. Fischl B, Sereno MI, Tootell RB, Dale AM. High-resolution intersubject averaging and a coordinate system for the cortical surface. Hum Brain Mapp. 1999;8:272–284.

14. Ségonne F, Pacheco J, Fischl B. Geometrically accurate topology-correction of cortical surfaces using nonseparating loops. IEEE Trans Med Imaging. 2007;26:518–529.

15. Ségonne F, Dale AM, Busa E, Glessner M, Salat D, Hahn HK, et al. A hybrid approach to the skull stripping problem in MRI. Neuroimage. 2004;22:1060–1075.

16. Reuter M, Schmansky NJ, Rosas HD, Fischl B. Within-subject template estimation for unbiased longitudinal image analysis. NeuroImage. 2012;61:1402–1418.

17. Reuter M, Rosas HD, Fischl B. Highly accurate inverse consistent registration: A robust approach. NeuroImage. 2010;53:1181–1196.

18. Kao C-Y, Hofer M, Sapiro G, Stem J, Rehm K, Rottenberg DA. A geometric method for automatic extraction of sulcal fundi. IEEE Trans Med Imaging. 2007;26:530–540.

19. Schaer M, Cuadra MB, Tamarit L, Lazeyras F, Eliez S, Thiran J-P. A surface-based approach to quantify local cortical gyrification. IEEE Trans Med Imaging. 2008;27:161–170.

20. Rouder JN, Speckman PL, Sun D, Morey RD, Iverson G. Bayesian t tests for accepting and rejecting the null hypothesis. Psychonomic Bulletin & Review. 2009;16:225–237.

21. Desikan RS, Ségonne F, Fischl B, Quinn BT, Dickerson BC, Blacker D, et al. An automated labeling system for subdividing the human cerebral cortex on MRI scans into gyral based regions of interest. NeuroImage. 2006;31:968–980.

22. Benjamini Y, Hochberg Y. Controlling the false discovery rate: a practical and powerful approach to multiple testing. J R Stat Soc B Met. 1995;57:289–300.

23. Hellerhoff I, Bernardoni F, Bahnsen K, King JA, Doose A, Pauligk S, et al. Serum neurofilament light concentrations are associated with cortical thinning in anorexia nervosa. Psychol Med. 2023:1–9.
